# Analysis of Lemon Verbena Polyphenol Metabolome and Its Correlation with Oxidative Stress under Glucotoxic Conditions in Adipocyte

**DOI:** 10.1021/acs.jafc.3c06309

**Published:** 2024-04-17

**Authors:** Mariló Olivares-Vicente, Noelia Sánchez-Marzo, María Herranz-López, Vicente Micol

**Affiliations:** † Instituto de Investigación, Desarrollo e Innovación en Biotecnología Sanitaria de Elche, Universidad Miguel Hernández (UMH), Elche 03202, Spain; ‡ CIBER: CB12/03/30038, Fisiopatología de la Obesidad y la Nutrición, CIBERobn, Instituto de Salud Carlos III (ISCIII), Madrid 28029, Spain

**Keywords:** cellular uptake, lemon verbena metabolite, adipocyte, oxidative stress, glucotoxicity, cellular metabolism

## Abstract

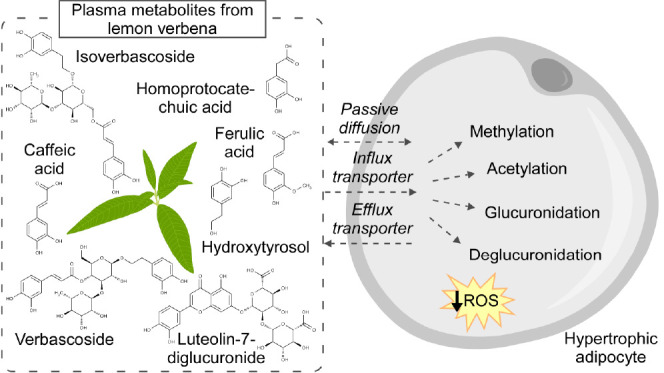

Lemon verbena has been shown to ameliorate obesity-related oxidative stress, but the intracellular final effectors underlying its antioxidant activity are still unknown. The purpose of this study was to correlate the antioxidant capacity of plasma metabolites of lemon verbena (verbascoside, isoverbascoside, hydroxytyrosol, caffeic acid, ferulic acid, homoprotocatechuic acid, and luteolin-7-diglucuronide) with their uptake and intracellular metabolism in hypertrophic adipocytes under glucotoxic conditions. To this end, intracellular ROS levels were measured, and the intracellular metabolites were identified and quantified by high-performance liquid chromatography with a diode array detector coupled to mass spectrometry (HPLC-DAD-MS). The results showed that the plasma metabolites of lemon verbena are absorbed by adipocytes and metabolized through phase II reactions and that the intracellular appearance of these metabolites correlates with the decrease in the level of glucotoxicity-induced oxidative stress. It is postulated that the biotransformation and accumulation of these metabolites in adipocytes contribute to the long-term antioxidant activity of the extract.

## 1 Introduction

Oxidative stress, defined as an overproduction of reactive oxygen species (ROS) in cells and tissues relative to antioxidant defense, plays a pathogenic role in the development of several chronic inflammatory diseases associated with obesity, such as insulin resistance, type 2 diabetes, and cardiovascular diseases.^1^ In this context, polyphenols from lemon verbena (*Lippia citriodora*), a digestive and anti-inflammatory plant used in folk medicine, have been associated with enough evidence to become an alternative way to alleviate the metabolic disturbances associated with obesity.^2−4^ Furthermore, several exercise training trials have demonstrated the potential use of lemon verbena extract as a nutraceutical to protect against oxidative damage and muscular injury caused by intense physical activity.^5^

The leaves of lemon verbena are rich in phenylpropanoids, iridoid glycosides, and flavonoids,^2,6^ and the bioactivity of this plant has primarily been attributed to its major component, namely verbascoside (also known as acteoside). In addition to its strong antioxidant power,^7^ this phenylpropanoid has been shown to exert anti-inflammatory and antilipogenic effects in hypertrophic adipocytes by upregulating the anti-inflammatory adipokine adiponectin and activating the energy sensor AMP-activated protein kinase.^4^ Despite this evidence, recent studies evaluating isolated fractions of lemon verbena extract obtained by semipreparative chromatography suggest that other compounds present in the extract, such as its isomer isoverbascoside or the flavonoid luteolin-7-diglucuronide, might also be potential contributors acting synergistically in the bioactivity of this plant.^2,8^

Furthermore, when evaluating the therapeutic effects of polyphenols, their bioavailability and biotransformation events should be considered to identify the most likely final effectors.^9^ In this regard, a previous bioavailability study in rats shows that verbascoside and isoverbascoside are the primary plasma metabolites after the ingestion of lemon verbena, but other minor metabolites have also been detected, such as phenylpropanoid derivatives (i.e., hydroxytyrosol, caffeic acid, ferulic acid, ferulic acid glucuronide, and homoprotocatechuic acid), as well as other phenolic compounds, iridoids, and flavone derivatives (i.e., luteolin-7-diglucuronide).^3^ Although the accumulation of some of these metabolites in target tissues, such as the brain, liver, and kidneys, has been reported,^10^ their distribution and the intracellular final effectors in adipose tissue are still unknown.

**Figure 1:**
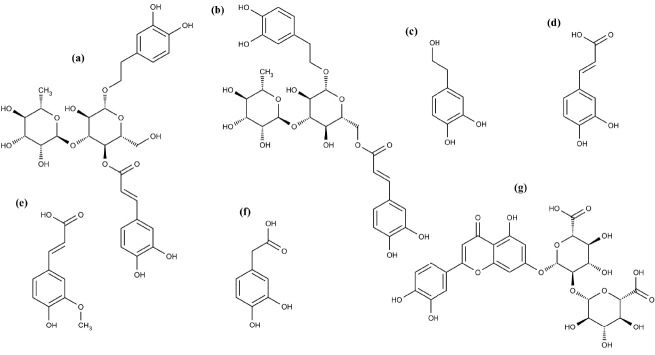
Molecular structure of seven plasma metabolites from lemon verbena: (a) verbascoside; (b) isoverbascoside; (c) hydroxytyrosol; (d) caffeic acid; (e) ferulic acid; (f) homoprotocatechuic acid; and (g) luteolin-7-diglucuronide.

To address this gap in knowledge, the aim of this study was to establish the antioxidant capacity of seven plasma metabolites of lemon verbena (i.e., verbascoside, isoverbascoside, hydroxytyrosol, ferulic acid, caffeic acid, homoprotocatechuic acid, and luteolin-7-diglucuronide) in a cell model of hypertrophic adipocytes (molecular structures in Figure 1). In addition to being the most abundant plasma metabolites identified after lemon verbena ingestion,^3^ verbascoside and isoverbascoside were selected for being potent antioxidant agents and modulating targets involved in energy expenditure and inflammation.^2,4^ Hydroxytyrosol, ferulic acid, caffeic acid, and homoprotocatechuic acid were selected as minor plasma metabolites of lemon verbena^3^ but with strong antioxidant power.^11−13^ Although little is known about their role in adipocytes,^14^ it is postulated that they might be relevant effectors of verbascoside and isoverbascoside since they are derived from the hydrolysis of these phenylpropanoids. Finally, luteolin-7-diglucuronide represents the only flavone derivative identified as a lemon verbena plasma metabolite that has been reported to modulate targets related to energy metabolism in adipocytes.^3,8^

Furthermore, the correlation of the decrease in intracellular ROS levels with the uptake and intracellular metabolism of these metabolites was addressed using high-performance liquid chromatography with a diode array detector coupled to electrospray ion trap mass spectrometry (HPLC-DAD-ESI-IT-MS). Using this experimental approach, we attempted to demonstrate that the plasma metabolites of lemon verbena are absorbed and metabolized by adipocytes through phase II reactions and that the intracellular appearance of these metabolites correlates with a decrease in glucotoxicity-associated oxidative stress in the cytoplasm.

## 2 Materials and Methods

### 2.1 Chemicals and Reagents

Standard verbascoside, hydroxytyrosol, and ferulic acid compounds were acquired from Extrasynthese (Genay, France). Isoverbascoside and luteolin-7-diglucuronide were purchased from PhytoLab (Vestenbergsgreuth, Germany). Caffeic acid and homoprotocatechuic acid were obtained from Sigma-Aldrich (St. Louis, MO, USA). Ethanol was provided by PanReac AppliChem GmbH (ITW Reagents, Darmstadt, Germany). Methanol (HPLC-MS grade) and glacial acetic acid were acquired from Fisher Scientific (Thermo Fisher Scientific, Waltham, MA, USA) and Labkem (Labbox, Barcelona, Spain), respectively. The water used in the HPLC-DAD-ESI-IT-MS analysis was purified using a Milli-Q system from Millipore (Bedford, MA, USA). Dulbecco’s modified Eagle’s medium (DMEM) and a mixture of penicillin/streptomycin were acquired from Gibco (Thermo Fisher Scientific). Calf and fetal bovine sera were obtained from Fisher Scientific. 3-Isobutyl-1-methylxanthine (IBMX), dexamethasone, insulin, dimethyl sulfoxide (DMSO), and phosphate-buffered saline (PBS) were provided by Sigma-Aldrich.

### 2.2 Model of Hypertrophic 3T3-L1 Adipocytes

The 3T3-L1 preadipocyte cell line (ATCC CL-173) was propagated and maintained in DMEM supplemented with 10% bovine calf serum, 100 U/mL penicillin, and 100 μg/mL streptomycin under optimal conditions (37 °C, 95% humidity, 5% CO_2_). Confluent preadipocytes were differentiated into adipocytes by incubating them in DMEM containing 25 mM glucose and supplemented with 10% fetal bovine serum and adipogenic agents (0.5 mM IBMX, 1 μM dexamethasone, and 10 μg/mL insulin) for 48 h. Afterward, the cells were maintained in high glucose DMEM supplemented with 10% fetal bovine serum and 10 μg/mL insulin for an additional 2 weeks. Under these glucotoxic conditions, nearly all cells become hypertrophic adipocytes with high intracellular levels of lipids and ROS, mainly as hydrogen peroxide (H_2_O_2_) content.^15,16^ The oxidative status of this cell model was evaluated as described in Supporting Information. Subsequently, the hypertrophic adipocytes were treated with the standard compounds at 25, 50, and 100 μM for 24 h or with 100 μM for 3, 12, and 24 h for the correlation analysis. Previously, pure compounds were dissolved in DMSO or water, depending on their solubilities, and reconstituted in culture medium for sterilization. The amount of DMSO used for the cells did not exceed 0.1%.

### 2.3 Quantification of ROS Generation by H_2_DCFDA Staining

After treating the hypertrophic adipocytes with lemon verbena metabolites, the intracellular ROS generation was analyzed by incubating the H_2_O_2_-sensitive fluorogenic dye 2′,7′-dichlorodihydrofluorescein diacetate (H_2_DCFDA, Sigma-Aldrich) at 40 μM for 30 min at 37 °C. Then, the cells were washed with PBS, and the fluorescence was measured at 495 nm excitation and 529 nm emission wavelengths using a cell imaging multimode microplate reader (Cytation 3, BioTek Instruments, Winooski, VT, USA). To dismiss any possible cytotoxic effect of the compounds, the Hoechst 33342 dye (Invitrogen, Thermo Fisher Scientific) was added at 4 μM, and stained nuclei were counted by taking microphotographs with a DAPI imaging filter cube.

### 2.4 Determination of MDA by HPLC with Fluorescence Detection

Malondialdehyde (MDA) was measured as a marker of lipid peroxidation in hypertrophic adipocytes treated with lemon verbena metabolites at 100 μM for 24 h.^17^ Briefly, the treated cells were washed and gathered with a scraper in PBS. Pooled cells were lysed by three cycles of freezing and thawing by sonication using an ice-cold bath sonicator and centrifuged at 16,000*g* for 15 min at 4 °C to collect the supernatant for MDA analysis. The protein concentration was analyzed by using the Pierce BCA Protein Assay Kit (Thermo Fisher Scientific). The supernatants were mixed with 0.05% butylhydroxytoluene (Sigma-Aldrich) in 99% ethanol to prevent oxidation reactions and 20% trichloroacetic acid (PanReac AppliChem GmbH) in 0.6 M HCl (VWR Chemicals, Radnor, Pennsylvania, USA) to precipitate protein. The mixtures were cooled on ice and centrifuged at 2,300*g* for 15 min at 4 °C. Then, the supernatants were mixed with preheated 0.6% thiobarbituric acid (TBA, Sigma-Aldrich) and heated at 97 °C for 30 min. After cooling, the samples were vigorously mixed with cooled *n*-butanol (Scharlab, Barcelona, Spain) and centrifuged at 18,000*g* for 3 min at 4 °C. The TBA-MDA complex contained in the upper phase was analyzed by HPLC using a Merck-Hitachi LaChrom system equipped with a model L-7485 fluorescence detector (Hitachi Instruments, Tokyo, Japan). The separation was carried out with a LiChrospher 100 RP-18 column (5 μm, 250 × 4 mm) (Merck, Darmstadt, Germany) using 50 mM potassium dihydrogen phosphate buffer (Merck) and methanol (60:40, v/v) as the mobile phase. The injection volume was 20 μL and the flow rate was 0.6 mL/min at room temperature. The TBA-MDA complex was monitored by fluorescence detection, with excitation at 515 nm and emission at 553 nm. The MDA content of the cell lysates was calculated from a standard curve prepared using different concentrations of TBA-MDA complex.

### 2.5 Sample Preparation for Metabolite Detection

After 0, 3, 12, and 24 h of incubation with 100 μM lemon verbena metabolites, the adipocytes were washed with PBS to remove the remaining compound from the extracellular side and gathered under cold ultrapure water. Pooled cells were lysed and centrifuged as mentioned above to collect supernatant and precipitate fractions as cytoplasmic and membrane fractions, respectively. The protein concentration in the cell lysates was analyzed using the Pierce BCA Protein Assay Kit. For HPLC analysis, the cytoplasmic and membrane fractions were subjected to protein precipitation using methanol:ethanol (50:50, v/v) at a proportion of 1:5 (sample–solvent), vortexed for 10 s, and centrifuged at 17,900*g* for 10 min at 4 °C. Lastly, the supernatants were collected and evaporated under nitrogen gas, dissolved in 120 μL of methanol:water (80:20, v/v), and stored at −80 °C until analysis.

### 2.6 HPLC-DAD-ESI-IT-MS Instruments and Conditions

An Agilent LC 1100 series system (Agilent Technologies, Inc., Palo Alto, CA, USA) equipped with a pump, autosampler, column oven, and DAD was used for the HPLC analysis of the cytoplasmic and membrane fractions. Aqueous acetic acid (0.5%, v/v) as solvent A and methanol as solvent B composed the mobile phase to elute the samples from a Poroshell 120 SB-C18 column (2.7 μm, 4.6 mm × 150 mm), which was protected by an InfinityLab Poroshell 120 SB-C18 guard column (2.7 μm, 4.6 mm × 5 mm). The following multistep linear gradient was established: 0 min, 5% B; 5 min, 5% B; 10 min, 15% B; 16 min, 17% B; 22 min, 20% B; 30 min, 30% B; 38 min, 33% B; 45 min, 100% B; and 50 min, 5% B. The initial conditions were maintained for 5 min to equilibrate the column for the next analysis, as indicated in the last step of the gradient. The flow rate was 0.5 mL/min, the injection volume was 10 μL, and the column temperature was set at 25 °C. The DAD monitored the spectrum range of 190–600 nm, and chromatograms at 280 and 340 nm were directly obtained.

The chromatographic system was coupled to a Bruker Esquire 3000 plus IT mass spectrometer equipped with an ESI source (Bruker Daltonics GmbsH, Bremen, Germany). An MS/MS analysis was performed in negative ionization mode considering the *m*/*z* range of 50–1400. The values of the other parameters were set as follows: capillary voltage, 2340 nV; dry temperature, 360 °C; dry gas flow, 9 L/min; nebulizing gas pressure, 45 psi; and fragmentation amplitude, 0.8 V. Data acquisition was performed with Data Analysis 4.0 software (Bruker Daltonics GmbsH).

### 2.7 Identification and Quantification of Metabolites

The separation of the metabolites was achieved using reversed-phase HPLC. The identification was performed by interpreting the molecular mass and product ions obtained by the mass spectrometer, taking into consideration the information provided from the literature. All MS results were compared with MS analysis of untreated adipocyte samples (cellular control) and of the solvents used in sample preparation (blank, without cellular material). Quantification was performed by preparing calibration curves with the seven standard compounds. Stock solutions were prepared at 100 μM in methanol:water (80:20, v/v) as the cellular samples, and six solutions at different concentrations were analyzed in triplicate for each standard. Peak areas were obtained from chromatograms at 340 nm for verbascoside, isoverbascoside, caffeic acid, ferulic acid, and luteolin-7-diglucuronide, whose linear regression ranged from 1.5625 to 100 μM. For hydroxytyrosol and homoprotocatechuic acid, solutions from 3.125 to 100 μM were plotted using chromatograms at 280 nm. The limit of detection (LOD) and limit of quantification (LOQ) for each compound were explored with the appropriate dilutions and estimated based on signal-to-noise ratios of less than 3 and 10, respectively. The LOD was designated when the metabolite was not observed in the DAD chromatograms, but traces were detected in the MS analysis.

### 2.8 Statistical Analysis

Statistical analyses were performed using GraphPad Prism ver. 7.04 (GraphPad Software, San Diego, CA, USA). The results are expressed as the mean ± standard deviation, unless otherwise indicated. Differences showing *p* < 0.05 were considered statistically significant. One-way analysis of variance (ANOVA) and Tukey’s post hoc test for multiple comparisons were used to assess differences in antioxidant capacity tests. Additionally, the Pearson coefficient was employed to assess the correlation between the antioxidant activity of incubated compounds and the intracellular appearance of their metabolites. Cellular experiments for antioxidant capacity and chromatographic analysis were independently performed three times.

## 3 Results and Discussion

### 3.1 Antioxidant Effect of Lemon Verbena Metabolites in Hypertrophic 3T3-L1 Adipocytes

To evaluate the antioxidant effect of lemon verbena metabolites on glucotoxicity-induced hypertrophic 3T3-L1 adipocytes, we first aimed to establish the potential contributors to oxidative stress in this cell model. Mature adipocytes with 10 days of differentiation were induced with 25 mM glucose for an additional 7 days to obtain hypertrophic adipocytes,^15^ and the generation of total ROS and superoxide anion (the main initial form of ROS in cells^18^) was assessed using fluorescent probes. As expected, long-term incubation with high glucose significantly induced ROS production, but not through superoxide anion formation, whose levels significantly increased in both mature and hypertrophic adipocytes only after induction with pyocyanin, a toxin released by *Pseudomonas aeruginosa* that produces a superoxide anion and ROS in the host cell cytoplasm^19^ (Figure S1). In accordance with this, H_2_O_2_ has been proposed as the primary contributor of ROS in the presence of high glucose in other cell types.^16^ In addition, the excess of glucose has been shown to generate ROS in 3T3-L1 adipocytes mainly through nicotinamide adenine dinucleotide phosphate (NADPH) oxidase 4,^20^ the only isoform expressed in adipocytes that primarily produces H_2_O_2_.^21^

**Figure 2:**
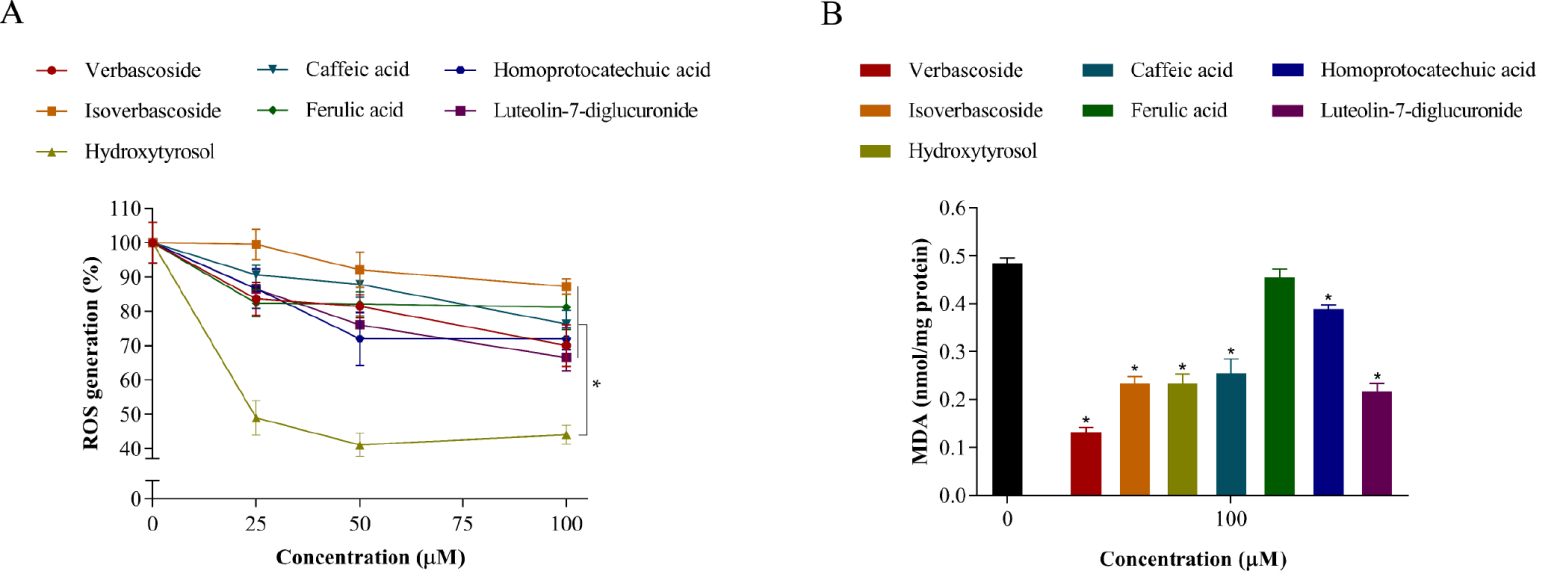
Effect of seven plasma metabolites from lemon verbena on oxidative stress in hypertrophic 3T3-L1 adipocytes. A) Intracellular ROS levels measured by H_2_DCFDA staining. The metabolites were added at 25, 50, and 100 μM for 24 h. * indicates significant differences between groups (*p* < 0.001). B) MDA content expressed in nmol of MDA per mg of protein. The metabolites were added at 100 μM for 24 h. * indicates significant differences compared to untreated hypertrophic adipocytes (*p* < 0.001).

For the reason mentioned above, the effect of lemon verbena metabolites on glucotoxicity-induced ROS generation in hypertrophic adipocytes was conveniently assessed throughout this study by using the cell-permeable probe H_2_DCFDA, which is rapidly oxidized to the fluorescent 2′,7′-dichlorofluorescein in the presence of ROS, predominantly H_2_O_2_. According to the cell count by Hoechst staining, none of the compounds showed a cytotoxic effect at the tested concentrations after incubation for 24 h (data not shown). All the compounds significantly reduced oxidative stress (Figure 2A). Among them, hydroxytyrosol was the most potent metabolite, reducing ROS levels to approximately 49% at the lowest concentration (25 μM, *p* < 0. 001) and maintaining the inhibitory effect at higher concentrations.

All the other compounds were less effective than hydroxytyrosol at reducing oxidative stress and exhibited a similar dose–response behavior, reaching a significant ROS reduction from 25 μM with verbascoside, ferulic acid, homoprotocatechuic acid, and luteolin-7-diglucuronide (16%, *p* < 0.001; 18%, *p* < 0.001; 13%, *p* < 0.01; and 13%, *p* < 0.05, respectively), from 50 μM with caffeic acid (12%, *p* < 0.05), and from 100 μM with isoverbascoside (13%, *p* < 0.001). These results confirm that, in addition to verbascoside, other plasma metabolites from lemon verbena have antioxidant power and may contribute to the biological activity of the extract, as previously suggested.^3^

The overproduction of ROS in cells may damage macromolecules such as lipids, causing lipid peroxidation.^22^ To test whether the antioxidant power of lemon verbena metabolites prevents lipid peroxidation in hypertrophic adipocytes, the cells were treated with the concentration at which all metabolites significantly reduced ROS (100 μM) and the content of MDA, the end product of this oxidative process,^22^ was measured. All metabolites except ferulic acid significantly reduced the content of this oxidative marker (Figure 2B), suggesting that some polyphenols are effective antioxidants in lipophilic environments.^7^

### 3.2 Correlation between Antioxidant Effects and Uptake of Lemon Verbena Metabolites in Hypertrophic Adipocytes

To identify the intracellular metabolites responsible for the antioxidant capacity, we performed a comparative study between the intracellular ROS decrease and the uptake of lemon verbena metabolites by hypertrophic adipocytes. Notably, we employed a concentration of 100 μM to aid in the identification of intracellular metabolites, considering it as the maximum nontoxic concentration at which the compounds demonstrated effectiveness. The analytical parameters for the seven compounds monitored by HPLC-DAD-ESI-IT-MS are summarized in Table S1. The chromatograms of the detected intracellular metabolites are shown in Figure S2, and the identification, retention time (RT), molecular mass, main MS/MS products, and quantification data for each peak are included in Tables S2–S8. In addition, the spectra of identified metabolites are shown in Figures S3–S9. In the case of verbascoside and isoverbascoside, metabolites were detected in both the membrane and cytoplasmic fractions (Table S9).

#### 3.2.1 Verbascoside

**Figure 3:**
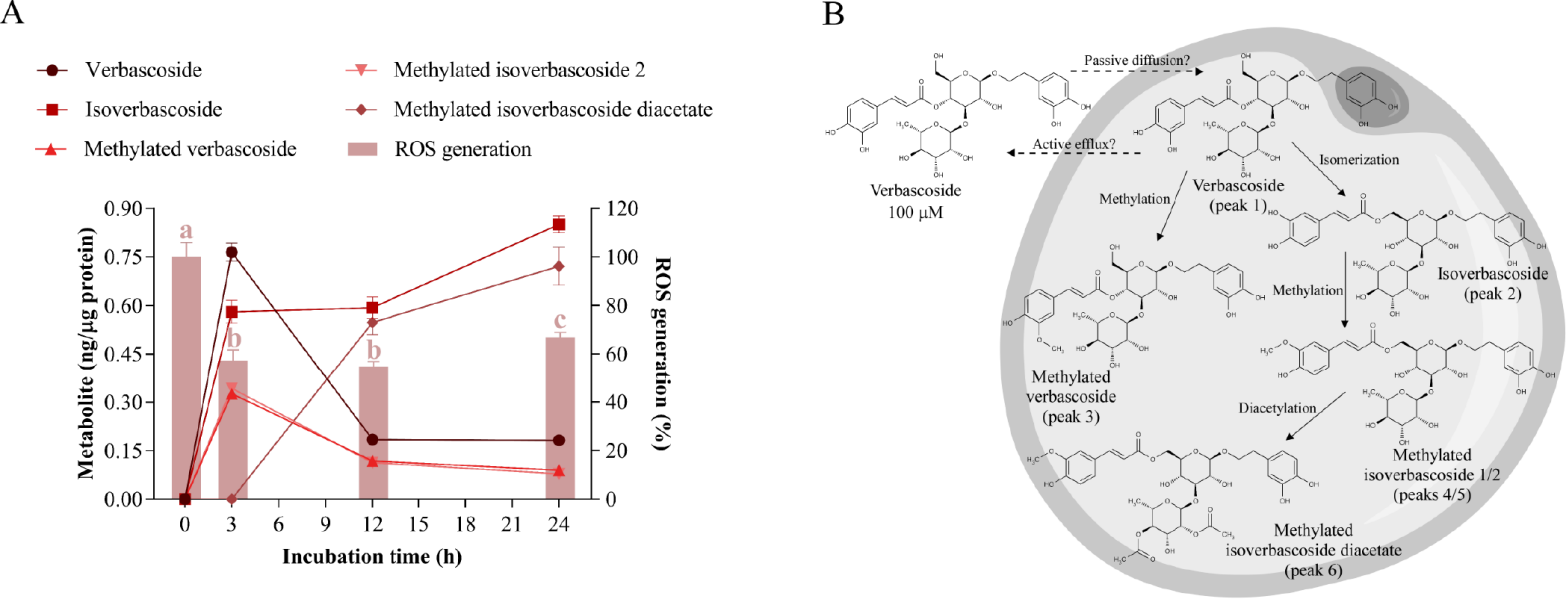
Cellular metabolism of verbascoside and its correlation with oxidative stress. A) Uptake and intracellular metabolism of verbascoside and its effect on ROS generation in hypertrophic 3T3-L1 adipocytes. Verbascoside was added at 100 μM for 3, 12, and 24 h of treatment. Different letters indicate significant differences between groups (*p* < 0.05). B) Scheme of the biotransformation pathways of verbascoside in the cytoplasm of a hypertrophic adipocyte.

Verbascoside is the primary phenylpropanoid glycoside present in lemon verbena and is structurally characterized by caffeoyl and hydroxytyrosol moieties bound to β-glucopyranoside through ester and glycosidic links, respectively, with rhamnose in sequence (1–3) to the glucose molecule (Figure 1a). When verbascoside was added at 100 μM to hypertrophic adipocytes, the maximum intracellular concentration of this compound (peak 1, *m*/*z* 623) was reached after 3 h of incubation (0.765 ± 0.027 ng/μg protein) and then declined dramatically at 12 h, maintaining a concentration of 0.182 ± 0.009 ng/μg at the end of the assay (24 h) (Figure 3A, Table S2). Conversely, its isomer isoverbascoside (peak 2, *m*/*z* 623) appeared in the cytoplasm at 0.580 ± 0.035 ng/μg after 3 h of incubation with verbascoside and progressively increased, reaching a maximum concentration at 24 h (0.850 ± 0.026 ng/μg).

In addition to isoverbascoside, four other intracellular metabolites derived from verbascoside were tentatively identified (Table S2): three methylated metabolites from verbascoside and isoverbascoside, which were named methylated verbascoside, methylated isoverbascoside 1, and methylated isoverbascoside 2 (peaks 3, 4, and 5, respectively, *m*/*z* 637), whose fragment ions at *m*/*z* 193 confirmed the methyl position in the caffeoyl moiety,^23^ and a methylated isoverbascoside derivative (peak 6, *m*/*z* 720) tentatively proposed as methylated isoverbascoside diacetate. Although the fragmentation pattern of this metabolite has not yet been reported, monoacetylated products of verbascoside have been identified in rat urine and feces, and they are 42 Da heavier than the parent compound.^23^

Methylated verbascoside and methylated isoverbascoside 2 appeared in the cytoplasm at 0.326 ± 0.006 and 0.342 ± 0.011 ng/μg, respectively, after 3 h of incubation with the standard and gradually decreased at the end of the experiment (Figure 3A, Table S2). Instead, methylated isoverbascoside 1 was detected below the LOQ at 24 h, and methylated isoverbascoside diacetate, not found at 3 h, was remarkably present after 12 and 24 h (0.547 ± 0.038 and 0.721 ± 0.058 ng/μg, respectively). Regarding the antioxidant effect, the maximum decrease in intracellular ROS levels was observed after 3 and 12 h of incubation (57 and 55% ROS generation, respectively) and then the oxidative stress rose slightly at the end of the assay (67% ROS generation) (Figure 3A).

These data indicate that verbascoside is rapidly absorbed in adipocytes. As previously suggested in the human intestinal Caco-2 cell model,^24^ verbascoside could cross the cell membrane through a passive diffusion mechanism but with active efflux probably mediated by transporters. However, the rapid decrease in the level of verbascoside in adipocytes may primarily be due to its high metabolism. The results show that verbascoside extensively isomerizes to isoverbascoside and that both undergo phase II reactions through the action of catechol-O-methyltransferases (COMTs) followed by acetyltransferases. It should be noted that methylation and acetylation increase the hydrophobicity of the metabolites, facilitating their diffusion through the cell membrane. However, the diacetylated product accumulated in the cytoplasm, suggesting that its excretion might depend on some efflux transporters (Figure 3B). Consistent with our data, the isomerization, methylation, and acetylation of verbascoside have been observed in vivo,^23^ suggesting that biotransformation can occur efficiently in the liver.^25^ In this sense, our data indicate that other cell types, such as adipocytes, can also metabolize this phenylpropanoid.

Because verbascoside was rapidly eliminated in hypertrophic adipocytes, the exhibited antioxidant effect may be primarily attributed to its isomer, isoverbascoside, that accumulated in the cytoplasm. This is consistent with its demonstrated radical scavenger activity.^2^ The findings of the Pearson correlation analysis support this idea, where, despite revealing nonsignificant negative correlations between the reduction in ROS levels and intracellular metabolites (Table S10), isoverbascoside had the correlation that most closely approached significance (*p* = 0.087). On the other hand, the diacetylated metabolite seems unlikely to contribute to this effect due to its late appearance. However, if this compound could exert any biological activity in adipocytes is unknown and deserves further attention.

It should be noted that verbascoside and its derivatives were significantly present in the membrane fractions (Table S9), confirming the reported affinity and distribution of this phenylpropanoid in lipid membranes.^26^ In fact, verbascoside was the most effective preventing lipid peroxidation (Figure 2B), and this effect has been related to its ability to interact with phospholipid membranes, acting as a radical scavenger in lipophilic environments.^7^

#### 3.2.2 Isoverbascoside

**Figure 4:**
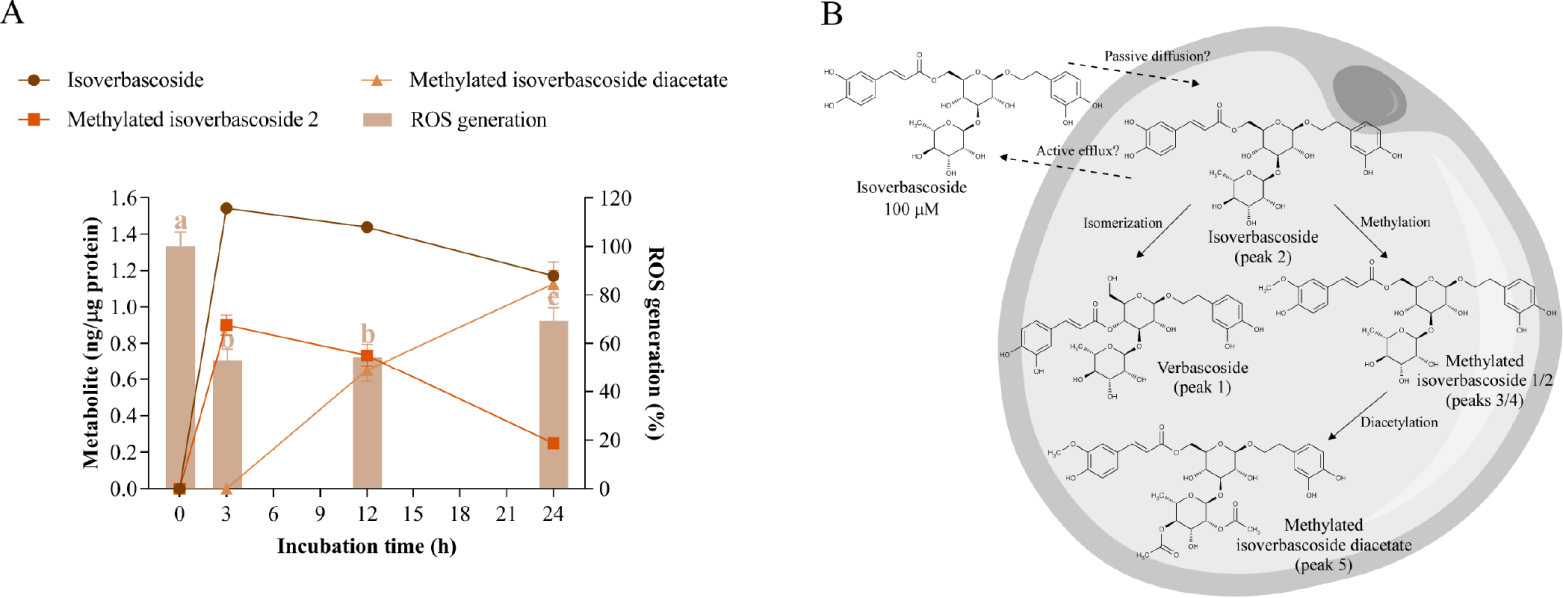
Cellular metabolism of isoverbascoside and its correlation with oxidative stress. A) Uptake and intracellular metabolism of isoverbascoside and its effect on ROS generation in hypertrophic 3T3-L1 adipocytes. Isoverbascoside was added at 100 μM for 3, 12, and 24 h of treatment. Different letters indicate significant differences between groups (*p* < 0.05). B) Scheme of the biotransformation pathways of isoverbascoside in the cytoplasm of a hypertrophic adipocyte.

Isoverbascoside is a structural isomer of verbascoside, in which the caffeoyl moiety is bound to β-glucopyranoside through the hydroxyl group of C6 instead of C4 (Figure 1b). Along with verbascoside, isoverbascoside is the primary plasma metabolite detected in rats after the ingestion of lemon verbena extract.^3^ When incubated at 100 μM in hypertrophic adipocytes, this compound (peak 2, *m*/*z* 623) achieved its maximum intracellular concentration at 3 h (1.541 ± 0.021 ng/μg) and then diminished slightly, reaching a concentration of 1.171 ± 0.076 ng/μg at the end of the experiment (Figure 4A, Table S3).

In addition, four other intracellular metabolites from isoverbascoside were tentatively identified in the cytoplasm of adipocytes: verbascoside (peak 1, *m*/*z* 623), methylated isoverbascoside 1 (peak 3, *m*/*z* 637), methylated isoverbascoside 2 (peak 4, *m*/*z* 637) and methylated isoverbascoside diacetate (peak 5, *m*/*z* 720) (Table S3). Methylated isoverbascoside 2 was detected at 0.900 ± 0.054 ng/μg after 3 h and gradually decreased throughout the assay, reaching 0.250 ± 0.015 ng/μg at 24 h (Figure 4A, Table S3). In addition, methylated isoverbascoside diacetate progressively appeared after 12 and 24 h (0.625 ± 0.062 and 1.127 ± 0.075 ng/μg, respectively). On the other hand, only traces of verbascoside were found, and methylated isoverbascoside 1 was detected below the LOQ.

Regarding the antioxidant effect, isoverbascoside achieved a maximum reduction in ROS levels after 3 and 12 h (53 and 54% ROS generation, respectively), increasing oxidative stress at the end of the experiment (69% ROS generation, Figure 4A). According to the Pearson correlation analyses (Table S10), the antioxidant behavior was strongly correlated to the uptake kinetics of isoverbascoside (*p* = 0.005), suggesting that this compound is the primary intracellular effector. As reported in hamster lung fibroblast cells,^27^ isoverbascoside could reduce oxidative stress in adipocytes by acting as a radical scavenger but also by modulating cellular antioxidant enzymes such as superoxide dismutase and catalase. Additionally, methylated isoverbascoside 2 showed a significant negative correlation between its intracellular appearance and ROS generation (*p* = 0.037), implying that this metabolite contributes to the antioxidant effect of isoverbascoside.

As also occurred after incubation with verbascoside, isoverbascoside is rapidly absorbed by adipocytes in a free form, probably by a passive diffusion mechanism, and it displays a high affinity for membranes (Table S9). Likewise, the biotransformation reactions (methylation and subsequent diacetylation) and the slow clearance of this metabolite in the cytoplasm of adipocytes were also confirmed (Figure 4B). In contrast, isomerization to verbascoside was much less favorable than that in the inverse direction. This finding may be explained by the instability of verbascoside under elevated pH values,^28^ such as physiological conditions, which underlines the importance of the tissue environment on the structure and bioactivity of polyphenols.

#### 3.2.3 Hydroxytyrosol

Hydroxytyrosol (4-[2-dihydroxyphenyl]ethanol) (Figure 1c) is the major phenolic component found in olive oil, either in the free form or as a part of the secoiridoid oleuropein, and it has been detected as a plasma metabolite of lemon verbena, probably derived from the hydrolysis of glycosidic bonds of verbascoside and isoverbascoside.^3^ When incubated at 100 μM, hydroxytyrosol was not detected in the cytoplasm of adipocytes at any time, but three new metabolites derived from the parent compound were identified and quantified (Table S4): two hydroxytyrosol glucuronides (peaks 1 and 2, *m*/*z* 329)^29^ and homovanillyl alcohol glucuronide (peak 3, *m*/*z* 343), whose fragment ion at *m*/*z* 167 confirmed a methoxy position in the benzene ring of the phenol moiety (homovanillyl alcohol).^29^

**Figure 5:**
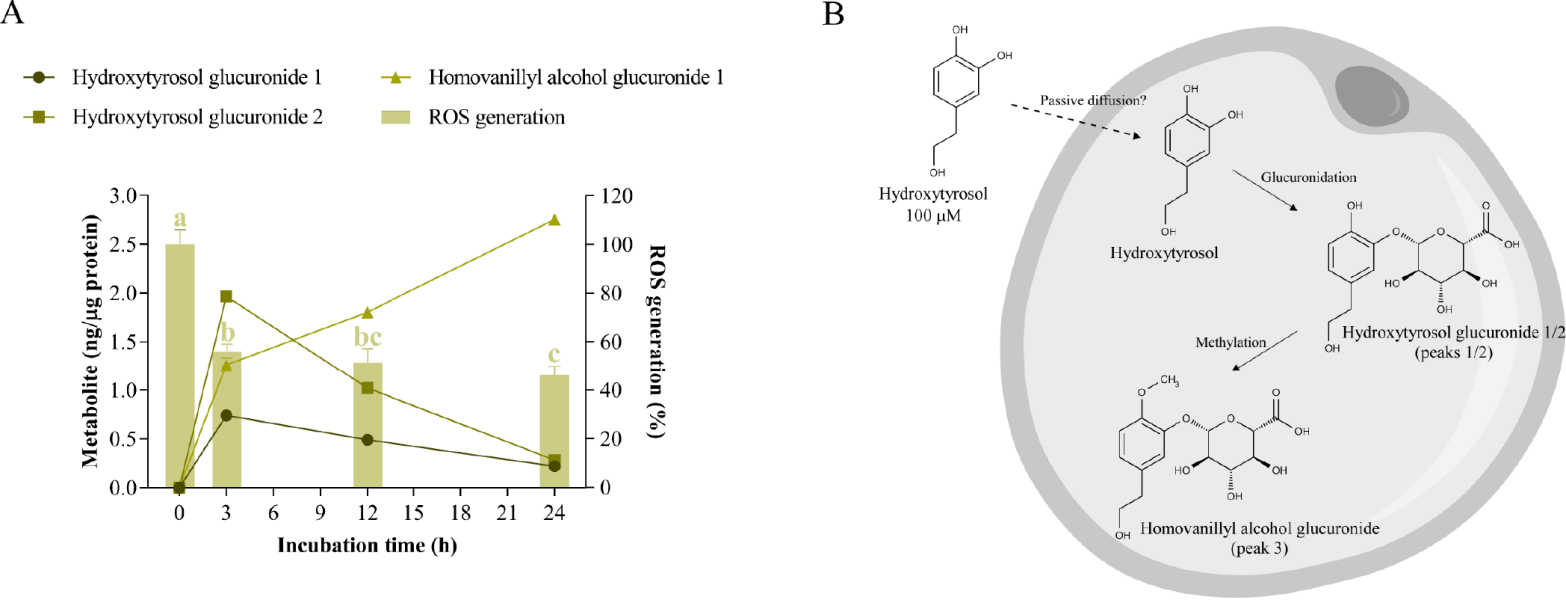
Cellular metabolism of hydroxytyrosol and its correlation with oxidative stress. A) Uptake and intracellular metabolism of hydroxytyrosol and its effect on ROS generation in hypertrophic 3T3-L1 adipocytes. Hydroxytyrosol was added at 100 μM for 3, 12, and 24 h of treatment. Different letters indicate significant differences between groups (*p* < 0.05). B) Scheme of the biotransformation pathways of hydroxytyrosol in the cytoplasm of a hypertrophic adipocyte.

First, the two hydroxytyrosol glucuronide isomers and homovanillyl alcohol glucuronide rapidly appeared in the cytoplasm at 3 h (0.742 ± 0.026, 1.967 ± 0.029, and 1.264 ± 0.069 ng/μg, respectively). However, both hydroxytyrosol glucuronides progressively disappeared throughout the experiment, while homovanillyl alcohol glucuronide accumulated, reaching a concentration of 2.754 ± 0.040 ng/μg at 24 h (Figure 5A, Table S4). Concurrently, oxidative stress diminished with incubation time, reaching its maximum reduction at the end of the experiment (46% ROS generation, Figure 5A). In concordance with this, the Pearson correlation coefficients confirmed a significant negative correlation between the latter metabolite and the reduction in ROS levels (*p* = 0.041) (Table S10).

Despite not detecting hydroxytyrosol in the cytoplasm of adipocytes, a previous study in Caco-2 cells demonstrated that this phenylethanoid crosses the cell membrane via passive diffusion.^30^ Since hydroxytyrosol uptake in adipocytes was not evaluated at incubation times of less than 3 h, we propose that this compound could rapidly diffuse in free form into the cells and be immediately conjugated by UDP-glucuronosyltransferases (UGTs) and O-methyltransferases. Furthermore, the absence of homovanillyl alcohol (methyl hydroxytyrosol) suggested that the parent compound is preferentially glucuronidated and subsequently methylated (Figure 5B). According to the above sequence of events, this metabolic pathway is also predominant in human hepatoma HepG2 cells after long incubation times.^31^ As reported with several flavonoids,^32^ this presence could occur because hydroxytyrosol induces UGT activity, favoring its own glucuronidation.

However, although methylation or glucuronidation could favor the excretion of metabolites, homovanillyl alcohol glucuronide accumulated in the cytoplasm of adipocytes. It has been reported that conjugated metabolites accumulate in certain tissues and form a metabolite pool for the release of active metabolites, which could explain this fact;^33^ but because no extracellular metabolites were assessed in this study, further research is needed to clarify the clearance of this metabolite.

Data on the in vitro antioxidant capacity of hydroxytyrosol metabolites compared to the parent compound are controversial,^34,35^ probably due to the fact that these metabolites require prior deglucuronidation to cross the cell membrane and exert their effects, as suggested previously in rat aorta rings and human red blood cells.^36,37^ Consistent with this property, hydroxytyrosol glucuronides have been shown to exert a comparable ability to that of the parent compound in protecting renal tubular epithelial cells against membrane oxidative damage, probably through direct antioxidant action.^38^

Notably, hydroxytyrosol, which is well-known as a potent radical scavenger, also induces the expression of glutathione-related antioxidant enzymes in HepG2 cells by enhancing the nuclear translocation of nuclear factor erythroid 2-related factor 2 (Nrf2) via phosphatidylinositol-3-kinase (PI3K)/protein kinase B (AKT) and extracellular signal-regulated kinase (ERK) pathways.^11^ According to this pathway, we propose that hydroxytyrosol may reverse oxidative stress in hypertrophic adipocytes through the same mechanism. However, whether glucuronide derivatives interact with intracellular targets once hydroxytyrosol is metabolized or, in contrast, whether hydroxytyrosol induces the cellular antioxidant defense system by interacting extracellularly with transmembrane receptors requires further research.

#### 3.2.4 Caffeic Acid

**Figure 6:**
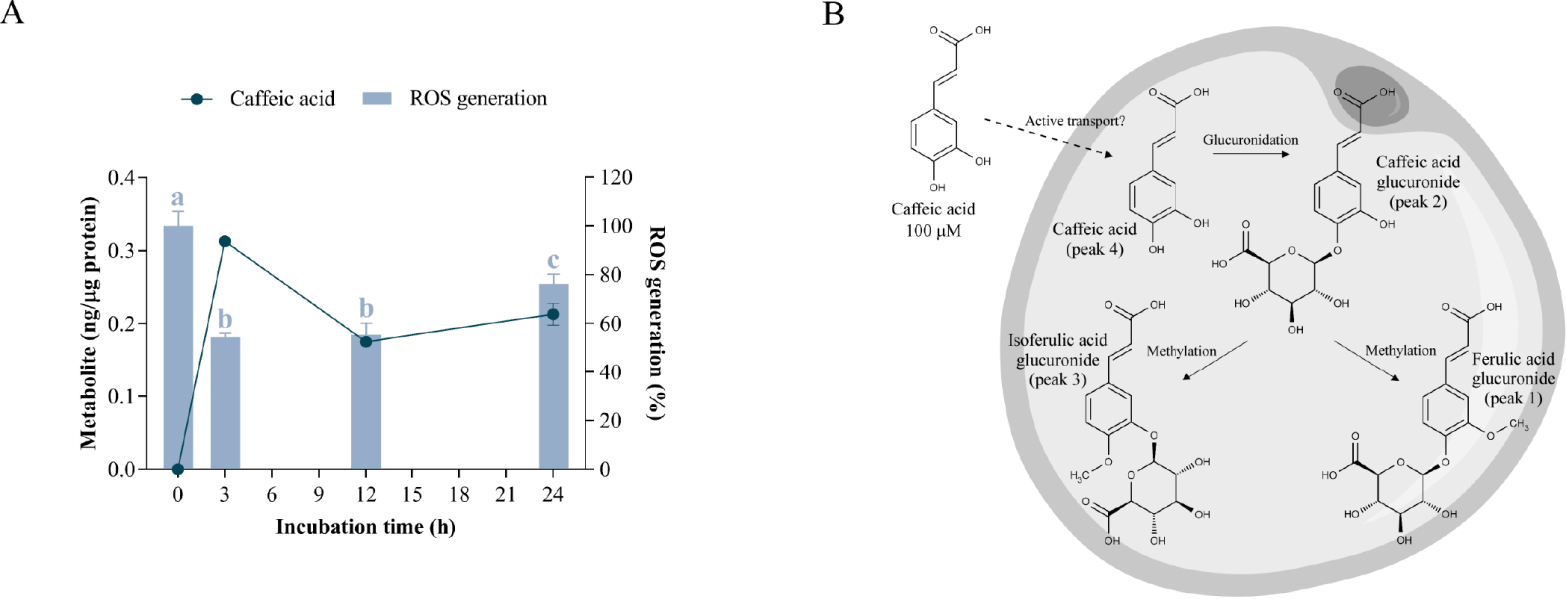
Cellular metabolism of caffeic acid and its correlation with oxidative stress. A) Uptake of caffeic acid and its effect on ROS generation in hypertrophic 3T3-L1 adipocytes. Caffeic acid was added at 100 μM for 3, 12, and 24 h of treatment. Different letters indicate significant differences between groups (*p* < 0.05). B) Scheme of the biotransformation pathways of caffeic acid in the cytoplasm of a hypertrophic adipocyte.

Caffeic acid (3,4-dihydroxycinnamic acid) (Figure 1d) is the most common hydroxycinnamic acid found in fruits, vegetables, cereals, and coffee, primarily as an ester with quinic acid (chlorogenic acid). Moreover, it represents a plasma metabolite of lemon verbena derived from the hydrolysis of glycosidic bonds of verbascoside and isoverbascoside.^3^ After incubation at 100 μM, caffeic acid (peak 4, *m*/*z* 179) was poorly absorbed by adipocytes, reaching a maximum concentration of 0.313 ± 0.006 ng/μg at 3 h and decreasing throughout the experiment (0.175 ± 0.007 and 0.213 ± 0.015 ng/μg after 12 and 24 h, respectively) (Figure 6A, Table S5). Despite the low intracellular concentration, caffeic acid exhibited a strong antioxidant effect on adipocytes, reducing oxidative stress to 55% after 3 and 12 h of incubation and increasing it at the end of the assay (76% ROS generation) (Figure 6A).

In addition, three new intracellular metabolites were detected below the LOD or LOQ (Table S5). They were identified as 3-methoxy caffeic acid 4-O-glucuronide (ferulic acid glucuronide, peak 1, *m*/*z* 369), caffeic acid glucuronide (peak 2, *m*/*z* 355), and 4-methoxy caffeic acid 3-O-glucuronide (isoferulic acid glucuronide, peak 3, *m*/*z* 369).^39^ The fragment ion at *m*/*z* 193 in peaks 1 and 3 corresponded to monohydroxycinnamic acid bearing a methoxy substituent at position 3 or 4 on the phenyl ring (ferulic acid or isoferulic acid, respectively), and the fragment ion at *m*/*z* 175 confirmed the glucuronidation at position 4 or 3. The longest RT of isoferulic acid compared to ferulic acid^40^ allowed us to identify peaks 1 and 3 as ferulic acid glucuronide and isoferulic acid glucuronide, respectively.

Among the seven lemon verbena metabolites assayed in this study, caffeic acid was the least absorbed by hypertrophic adipocytes. The poor absorption of caffeic acid is consistent with previous studies in Caco-2 and HepG2 cells.^41,42^ In fact, this hydroxycinnamate is transported in enterocytes via paracellular diffusion and, to a lesser extent, by the monocarboxylic acid transporter (MCT).^42^ Previous studies have demonstrated that MCT1 and MCT4 are expressed in mouse and human adipocyte cell lines and that their expression can be induced during differentiation or by hypoxia or cold exposure,^43,44^ suggesting a low expression level in our in vitro model of hypertrophic cells that explains the poor absorption of caffeic acid. Nevertheless, this characteristic should be considered with caution and deserves further investigation.

Caffeic acid was found to be largely intact in the cytoplasm of adipocytes with only traces of glucuronide and methyl glucuronide derivatives. Furthermore, the absence of methylated caffeic acid (ferulic acid) indicated that glucuronidation occurred prior to methylation (Figure 6B), as observed with hydroxytyrosol (Figure 5B). Consistent with our results, caffeic acid accumulates little in the cytoplasm of HepG2 cells but exhibits a higher rate of metabolism,^41^ probably due to the higher activity and isoform diversity of phase II drug-metabolizing enzymes in hepatocytes.^45^ Moreover, caffeic acid has been shown to diminish oxidative stress-induced hepatotoxicity by enhancing the expression of detoxification enzymes such as heme oxygenase-1 and glutamate-cysteine ligase via the ERK/Nrf2 pathway.^46^ Despite the lack of statistical significance in the Pearson correlation analysis (*p* = 0.074) (Table S10), the substantial accumulation of free caffeic acid in the cytoplasm suggests that this metabolite is the intracellular effector responsible for reducing ROS in hypertrophic adipocytes, probably acting as a direct radical scavenger^12^ but also modulating the ERK/Nrf2 pathway.

#### 3.2.5 Ferulic Acid

**Figure 7:**
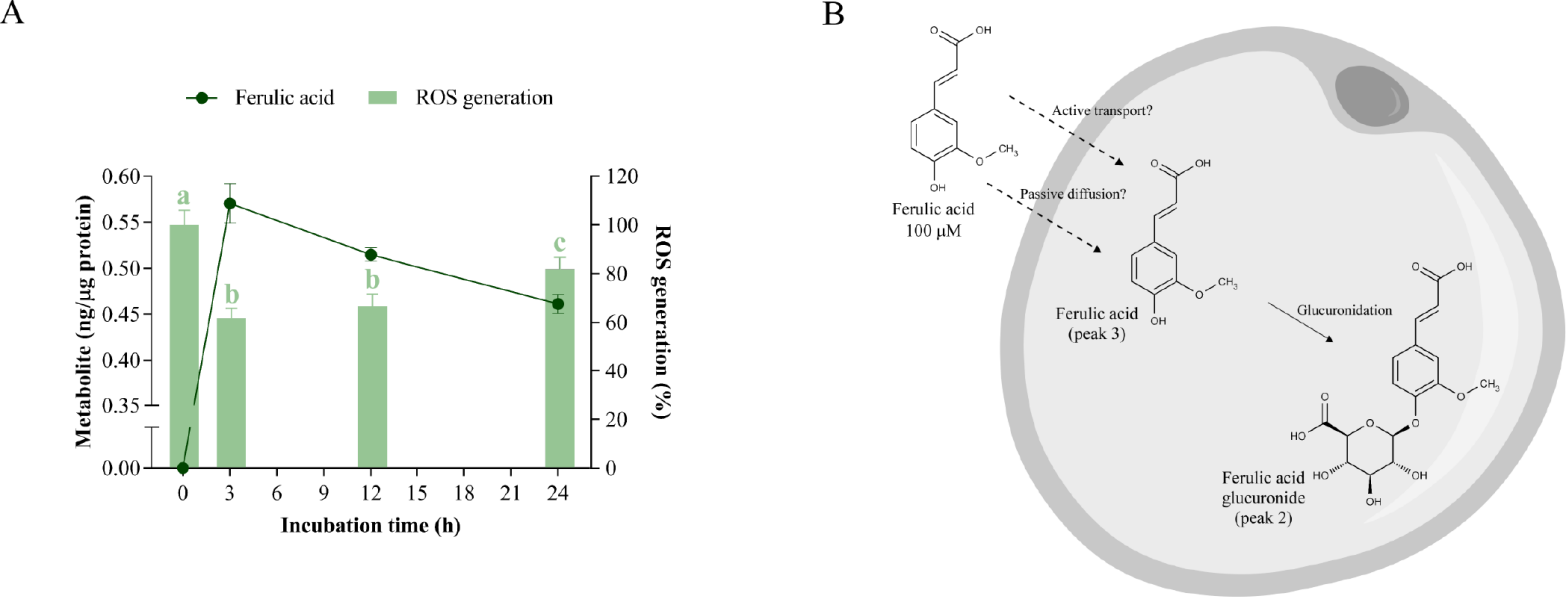
Cellular metabolism of ferulic acid and its correlation with oxidative stress. A) Uptake of ferulic acid and its effect on ROS generation in hypertrophic 3T3-L1 adipocytes. Ferulic acid was added at 100 μM for 3, 12, and 24 h of treatment. Different letters indicate significant differences between groups (*p* < 0.05). B) Scheme of the biotransformation pathways of ferulic acid in the cytoplasm of a hypertrophic adipocyte.

Ferulic acid (4-hydroxy-3-methoxycinnamic acid) (Figure 1e), along with caffeic acid, is an abundant hydroxycinnamic acid in foods and has been detected as a plasma metabolite of lemon verbena, probably from the 3-O-methylation of the caffeic acid moiety after the hydrolysis of glycosidic bonds of verbascoside and isoverbascoside.^3,47^ When ferulic acid was added to adipocytes at 100 μM, the maximum intracellular concentration of this compound (peak 3, *m*/*z* 193) was reached after 3 h of incubation (0.570 ± 0.022 ng/μg) and then slightly decreased, accumulating 0.461 ± 0.010 ng/μg at 24 h (Figure 7A, Table S6). Moreover, the antioxidant behavior was correlated with the intracellular accumulation of ferulic acid, showing the lowest ROS levels at 3 and 12 h (62 and 67% ROS generation, respectively) and increasing throughout the experiment (82% ROS generation at 24 h, Figure 7A).

In addition, two new metabolites below the LOD or LOQ were detected in the cytoplasm of adipocytes after incubation with ferulic acid (Table S6): unknown (peak 1, *m*/*z* 500) and ferulic acid glucuronide (peak 2, *m*/*z* 369). The moderate uptake and metabolism of ferulic acid are consistent with those reported in in vitro hepatic and colonic epithelium models,^41,48^ in which glucuronidation was the primary metabolic transformation of this hydroxycinnamate. Our data also indicate that ferulic acid is more extensively absorbed than caffeic acid, as observed in HepG2 cells.^41^ Previously, MCT was reported to mediate the transepithelial transport of ferulic acid,^49^ as is the case for caffeic acid,^42^ but with a higher affinity due to the presence of the methoxy substituent.^50^ Likewise, another study has shown that ferulic acid is mostly transported by passive diffusion in the colonic epithelium,^48^ a mechanism that is facilitated by the higher hydrophobicity of this compound compared to caffeic acid (Figure 7B).

According to our data, free ferulic acid was the predominant form that accumulated in the cytoplasm of adipocytes, indicating that it acts as the intracellular effector responsible for decreasing glucotoxicity-induced oxidative stress. This observation is further supported by the Pearson correlation coefficient (*p* = 0.032) (Table S10). As in the case of many polyphenols, the specific mechanism of the antioxidant effect of ferulic acid seems to be complex since it is able to scavenge free radicals, enhance antioxidant enzymes, and inhibit pro-oxidant enzymes.^51^ In this regard, ferulic acid suppressed ROS generation in rat vascular smooth muscle cells exposed to H_2_O_2_ by increasing the activity of antioxidant enzymes (superoxide dismutase, catalase, and glutathione peroxidase) and inhibiting the expression and activity of NADPH oxidase.^52^

#### 3.2.6 Homoprotocatechuic Acid

**Figure 8:**
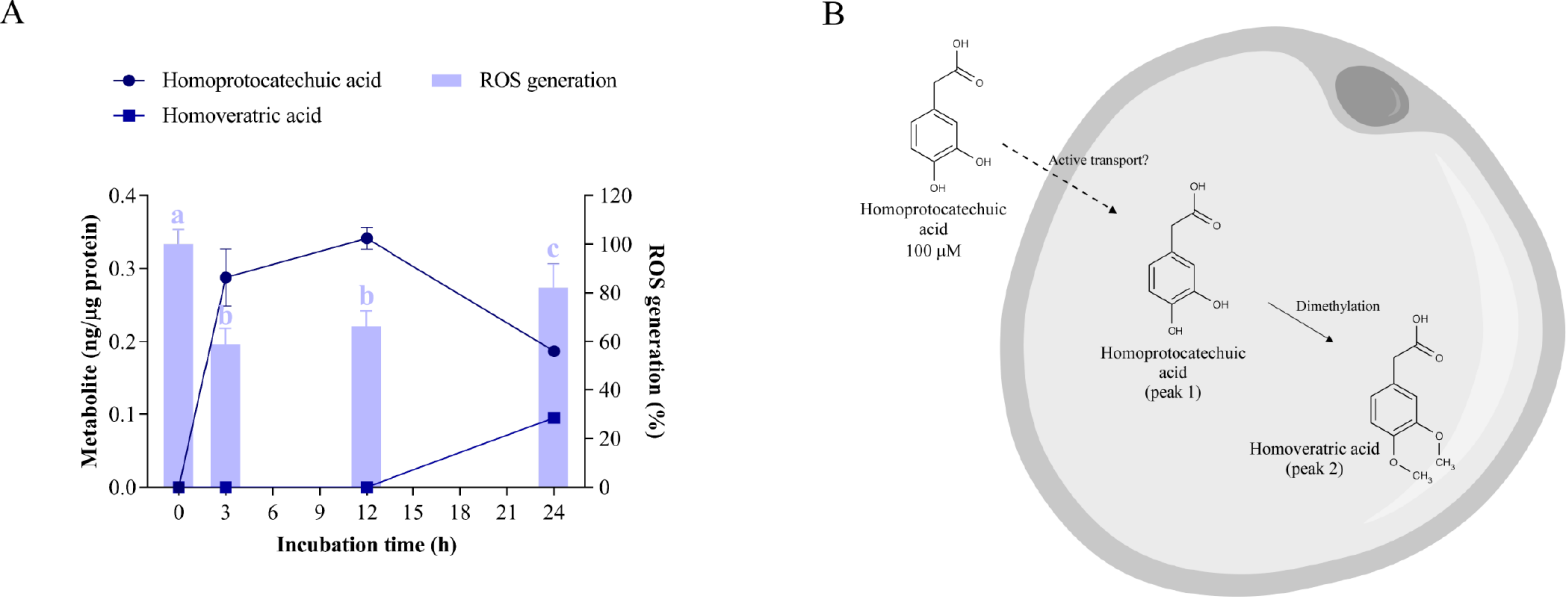
Cellular metabolism of homoprotocatechuic acid and its correlation with oxidative stress. A) Uptake and intracellular metabolism of homoprotocatechuic acid and its effect on ROS generation in hypertrophic 3T3-L1 adipocytes. Homoprotocatechuic acid was added at 100 μM for 3, 12, and 24 h of treatment. Different letters indicate significant differences between groups (*p* < 0.05). B) Scheme of the biotransformation pathways of homoprotocatechuic acid in the cytoplasm of a hypertrophic adipocyte.

Homoprotocatechuic acid (3,4-dihydroxyphenylacetic acid, DOPAC) (Figure 1f) is a neuronal metabolite of dopamine but is also produced through the metabolism of certain phenolic acids and flavonoids in the gastrointestinal tract. It has been identified in rat plasma after lemon verbena ingestion,^3^ probably through the biotransformation of caffeic acid or hydroxytyrosol.^53,54^ When added at 100 μM to hypertrophic adipocytes, homoprotocatechuic acid (peak 1, *m*/*z* 167)^55^ was detected intracellularly at 0.288 ± 0.039 ng/μg after 3 h of incubation; it reached its maximum concentration (0.342 ± 0.015 ng/μg) at 12 h and decreased to a final concentration of 0.187 ± 0.006 ng/μg at the end of the assay (Figure 8A, Table S7). Concurrently, intracellular ROS levels decreased strongly at 3 and 12 h (59% and 66% ROS generation, respectively), restoring with the clearance of homoprotocatechuic acid at 24 h (82% ROS generation, Figure 8A).

In addition, a new intracellular metabolite tentatively identified as homoveratric acid (3,4-dimethoxyphenylacetic acid, peak 2, *m*/*z* 195)^56^ appeared after incubation with homoprotocatechuic acid, reaching a concentration of 0.095 ± 0.005 ng/μg at 24 h (Figure 8A, Table S7). These data indicate that homoprotocatechuic acid is poorly absorbed and accumulated in adipocytes, similar to caffeic acid (Figure 6A). To our knowledge, this is the first time that the cellular uptake of homoprotocatechuic acid has been explored, but the structural similarity to that of caffeic acid suggests that its influx transport could require the involvement of some transporters.^50^ Once in the cytoplasm, homoprotocatechuic acid was slowly metabolized to homoveratric acid, suggesting that methylation is the primary clearance pathway for this phenolic acid in adipocytes (Figure 8B).

In addition, the decrease in ROS generation might be exclusively attributed to homoprotocatechuic acid since the ROS levels increased with the clearance of this compound, as evidenced by the Pearson correlation coefficient (*p* = 0.028) (Table S10). In this context, other authors have shown that homoprotocatechuic acid is a catabolite from quercetin glycosides that acts as a free radical scavenger but also as an indirect antioxidant by inducing phase II detoxification gene expression, such as glutathione *S*-transferase.^13^ Moreover, the presence of homoveratric acid in adipocytes did not contribute to the antioxidant activity. It is proposed that the methylation of both free 3- and 4-hydroxyl groups eliminates the chemical antioxidant capacity of the parent compound, consistent with a previous study in which 3,4-dimethoxycaffeic acid (caffeic acid without its hydroxyl groups) exhibited very little antioxidant capacity compared to caffeic acid or ferulic acid (monomethylated caffeic acid).^57^

#### 3.2.7 Luteolin-7-diglucuronide

**Figure 9:**
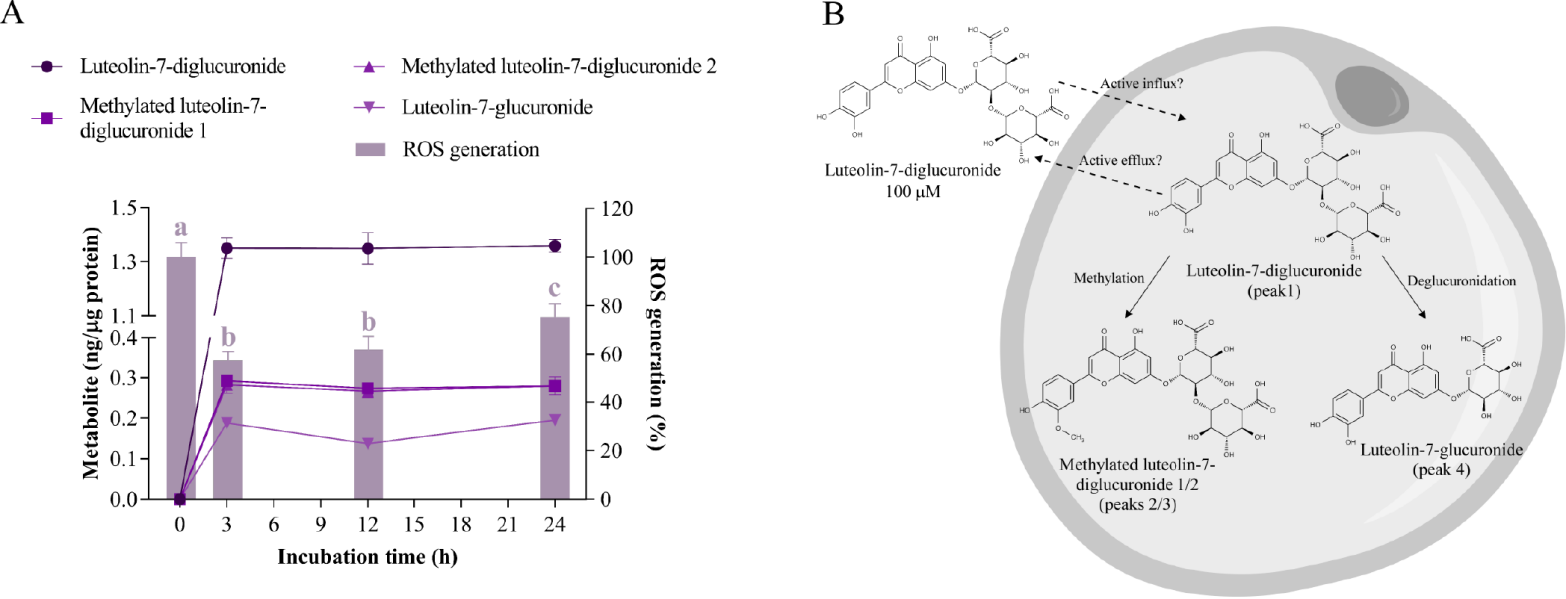
Cellular metabolism of luteolin-7-diglucuronide and its correlation with oxidative stress. A) Uptake and intracellular metabolism of luteolin-7-diglucuronide and its effect on ROS generation in hypertrophic 3T3-L1 adipocytes. Luteolin-7-diglucuronide was added at 100 μM for 3, 12, and 24 h of treatment. Different letters indicate significant differences between groups (*p* < 0.05). B) Scheme of the biotransformation pathways of luteolin-7-diglucuronide in the cytoplasm of a hypertrophic adipocyte.

Luteolin-7-diglucuronide (luteolin-7-O-[β-glucuronosyl(1→2)-β-glucuronide]) (Figure 1g) is present in lemon verbena extract and represents one of the primary flavone derivatives detected in rat plasma after the ingestion of the extract, which may appear through the absorption of the intact compound in the gut or after its hydrolysis to luteolin and subsequent conjugation to diglucuronide during its passage through the enterocytes.^3^ When incubated at 100 μM in hypertrophic adipocytes, luteolin-7-diglucuronide (peak 1, *m*/*z* 637)^58^ appeared rapidly in the cytoplasm, reaching a concentration of 1.350 ± 0.038 ng/μg after 3 h that was maintained until the end of the assay (Figure 9A, Table S8).

Furthermore, three new intracellular metabolites were tentatively identified after incubating with the standard (Table S8): two isomers of methylated luteolin-7-diglucuronide (peaks 2 and 3, *m*/*z* 651), whose fragment ions at *m*/*z* 299 may correspond to chrysoeriol (3′-O-methyl luteolin) or diosmetin (4′-O-methyl luteolin),^59^ and luteolin-7-glucuronide (peak 4, *m*/*z* 461).^60^ After 3 h, the two methylated isomers appeared in the cytoplasm at 0.292 ± 0.004 and 0.283 ± 0.021 ng/μg and luteolin-7-glucuronide was detected at 0.188 + 0.012 ng/μg. As with the parent compound, the concentrations of the new metabolites were constant throughout the experiment (Figure 9A,Table S8). Regarding the antioxidant effect, the maximum decrease in intracellular ROS levels was observed after 3 and 12 h (58 and 62% ROS generation, respectively), and then oxidative stress increased slightly at the end of the assay (75% ROS generation) (Figure 9A).

Due to the high polarity of glucuronide conjugates, the rapid intracellular appearance of luteolin-7-diglucuronide suggests the existence of a transporter that mediates the entry of this compound into the cell. In this regard, several transporters for flavonoids have been previously described.^61^ In particular, two members of the organic anion transporting polypeptide (OATP) family (i.e., OATP1B1 and 1B3) have been shown to actively take up luteolin-3′-glucuronide in liver cells.^62^ Additionally, the transport of other flavonoid glucuronides, such as quercetin-3-glucuronide and quercetin-7-glucuronide, has been proposed to occur through an unidentified transporter similar to OATP2.^63^ However, flavonoid transporters have not yet been discovered in adipocytes.

Although the biotransformation of luteolin-7-diglucuronide is unknown, its aglycone luteolin has been shown to undergo extensive metabolism in vivo. Luteolin monoglucuronides and methylated luteolin glucuronides have been found in rat plasma after the administration of luteolin.^64^ Our data show that luteolin-7-diglucuronide is also subject to the action of COMTs in adipocytes, yielding chrysoeriol-7-diglucuronide and diosmetin-7-diglucuronide. In addition, the formation of luteolin monoglucuronide suggests the presence of β-glucuronidase activity in these cells (Figure 9B). This enzymatic activity was also observed in hypertrophic 3T3-L1 adipocytes after incubation with quercetin-3-glucuronide^65^ and seemed to be enhanced under certain conditions of cell damage, as occurs in inflammation.^66^

Furthermore, no clearance of luteolin metabolites was observed during the incubation time of this experiment, suggesting an inhibition of efflux transporters. Some flavonoids can act as the potent inhibitors of efflux transporters, such as breast cancer resistance protein (BCRP).^67^ Indeed, BCRP inhibition has been shown to reduce the excretion of luteolin monoglucuronides, favoring the formation of diglucuronide in HeLa cells overexpressing UGT1A9.^68^ However, further research should be conducted to clarify the specific mechanism underlying the cellular excretion of luteolin glucuronides.

Based on the Pearson correlation coefficients (Table S10), the intracellular concentrations of luteolin-7-diglucuronide and its methylated isomers (1 and 2) exhibited a negative correlation with ROS levels (*p* = 0.042, *p* = 0.037 and *p* = 0.043, respectively). Considering that luteolin-7-diglucuronide was the predominant intracellular metabolite in hypertrophic adipocytes, it is proposed to be the primary responsible for the decrease in ROS generation, with a modest contribution from its methylated metabolites. The in vitro antioxidant capacity of this flavonoid diglucuronide has been previously reported,^2,69^ but little is known about its pharmacological activity in vivo. Other authors have shown cardioprotective effects in mice,^70^ proposing that the prevention of isoproterenol-induced myocardial injury and fibrosis is due, in part, to the reduction of ROS generation by inhibiting the expression of NADPH oxidase subunits in the heart. Considering this, luteolin-7-diglucuronide emerges as a potential protective agent against oxidative-stress-associated metabolic disorders.

The overall results indicate that phenolic metabolites from lemon verbena are rapidly absorbed in free form by hypertrophic 3T3-L1 adipocytes and undergo phase II and, to a lesser extent, phase I reactions probably through the action of methyltransferase, UGT, acetylase, and β-glucuronidase enzymes. In particular, verbascoside, isoverbascoside, hydroxytyrosol, and luteolin-7-diglucuronide are absorbed more extensively than phenolic acids, which probably favors their higher intracellular metabolism. In addition, glucuronide conjugations seem to be predominant on phenylethanoids and phenolic acids, while polyphenols with more complex structures are preferentially methylated. Accordingly, these metabolites, either in free form or as conjugated metabolites, are intracellular effectors that decrease glucotoxicity-induced oxidative stress. We propose that isoverbascoside, homovanillyl alcohol glucuronide, caffeic acid, ferulic acid, homoprotocatechuic acid, and luteolin-7-diglucuronide are the final effectors that exert these antioxidant effects, probably as radical scavengers or interacting with antioxidant enzymes in hypertrophic adipocytes. It should be noted that the antioxidant effect exhibited by the seven metabolites evaluated was maintained, even after 24 h. Although the concentrations of the major metabolites found in plasma of rats after the ingestion of lemon verbena extract are within the nanomolar range,^3^ it is postulated that the slow clearance of these metabolites in cells might favor their intracellular accumulation and, consequently, their long-term antioxidant effects in tissues. This is particularly relevant for the formulation of dietary supplements, as they are intended for daily consumption to ensure effectiveness.

In conclusion, these data provide evidence of how the bioactivity of polyphenols depends on their uptake, metabolism, and accumulation in target cells. In particular, these results offer a better understanding of their potential molecular mechanisms and, consequently, of the therapeutic effect of lemon verbena in ameliorating obesity-induced oxidative stress.

## Supporting Information Available

Supplementary materials and methods; effect of glucotoxicity on oxidative stress in hypertrophic 3T3-L1 adipocytes (Figure S1); linear regression data, LOD, and LOQ of the standard compounds monitored by DAD (Table S1); representative chromatograms of the intracellular metabolites detected by HPLC-DAD-ESI-IT-MS in hypertrophic adipocytes (Figure S2); quantification of intracellular metabolites characterized by HPLC-DAD-ESI-IT-MS in negative mode after incubation with verbascoside, isoverbascoside, hydroxytyrosol, caffeic acid, ferulic acid, homoprotocatechuic acid, and luteolin-7-diglucuronide in hypertrophic 3T3-L1 adipocytes (Tables S2–S8, respectively); MS spectra of intracellular metabolites described in Tables S2–S8 (Figures S3–S9, respectively); concentration of intracellular metabolites in the membrane fractions after incubation with verbascoside or isoverbascoside in hypertrophic 3T3-L1 adipocytes (Table S9). Pearson correlation coefficients between the ROS levels exhibited in hypertrophic 3T3-L1 adipocytes after incubation with the seven lemon verbena compounds and the concentration of their intracellular metabolites over time (Table S10) (PDF)

## Abbreviations:

BCRP: breast cancer resistance protein
COMT: catechol-O-methyltransferase
DMEM: Dulbecco’s modified Eagle’s medium
DMSO: dimethyl sulfoxide
H_2_DCFDA: 2′,7′-dichlorodihydrofluorescein diacetate
H_2_O_2_: hydrogen peroxide
HPLC-DAD-ESI-IT-MS: high-performance liquid chromatography with a diode array detector coupled to electrospray ion trap mass spectrometry
IBMX: 3-isobutyl-1-methylxanthine
LOD: limit of detection
LOQ: limit of quantification
MCT: monocarboxylic acid transporter
MDA: malondialdehyde
NADPH: nicotinamide adenine dinucleotide phosphate
Nrf2: nuclear factor erythroid 2-related factor 2
OATP: organic anion transporting polypeptide
PBS,: phosphate-buffered saline
ROS: reactive oxygen species
RT: retention time
TBA: thiobarbituric acid
UGT: UDP-glucuronosyltransferases
