# Supplementary material for: Analysis of Lemon Verbena Polyphenol Metabolome and Its Correlation with Oxidative Stress under Glucotoxic Conditions in Adipocyte
Source: J Agric Food Chem. 2024 Apr 17;72(17):9768–81. doi: 10.1021/acs.jafc.3c06309 (PMC11066870; doi:10.1021/acs.jafc.3c06309)
Supplement: Supplementary file 1 — jf3c06309_si_001.pdf [file jf3c06309_si_001.pdf]

Supporting Information

Analysis of lemon verbena polyphenol metabolome and its correlation with oxidative stress under glucotoxic conditions in adipocyte

Mariló Olivares-Vicente [a], Noelia Sánchez-Marzo [a], María Herranz-López [a,*,1] and Vicente Micol [a,b,1]

[a] Instituto de Investigación, Desarrollo e Innovación en Biotecnología Sanitaria de Elche, Universidad Miguel Hernández (UMH), 03202 Elche, Spain; maria.olivaresv@umh.es (M.O.-V.); n.sanchez@umh.es (N.S.-M); mherranz@umh.es (M.H.-L); vmicol@umh.es (V.M)

[b] CIBER: CB12/03/30038, Fisiopatología de la Obesidad y la Nutrición, CIBERobn, Instituto de Salud Carlos III (ISCIII), 28029 Madrid, Spain

* Corresponding author: mherranz@umh.es

[1] These authors share co-senior authorship

Supplementary Materials and Methods

Evaluation of the oxidative status of glucotoxicity-induced hypertrophic 3T3-L1 adipocytes

To evaluate the oxidative status of hypertrophic adipocytes induced by high glucose, total ROS and superoxide anion levels of mature and hypertrophic 3T3-L1 adipocytes obtained after 10 and 17 days of differentiation, respectively, with 25 mM glucose were determined. As a positive control, the ROS inducer pyocyanin was incubated in both mature and hypertrophic adipocytes at 20 µM for 24 h prior to measurement. Then, total ROS and superoxide anion levels were determined using the ROS/Superoxide Detection Assay Kit (Abcam, Cambrigde, UK) according to the manufacturer's instructions. The fluorescence was measured at 550 nm excitation and 610 nm emission wavelengths using the Cytation 3 reader.

Supplementary Figures and Tables

Figure S1. Effect of glucotoxicity on oxidative stress in hypertrophic 3T3-L1 adipocytes. Hypertrophy of mature adipocytes was induced by incubating with 25 mM glucose for 7 days. As a positive control of ROS generation in both mature and hypertrophic adipocytes, pyocyanin was incubated at 20 µM for 24 h prior to analysis. Then, total ROS and superoxide anion levels were measured by fluorescent probes. *, ** and *** indicate significant differences compared to mature adipocytes incubated with high glucose alone ($p < 0.05$, $p < 0.01$ and $p < 0.001$, respectively).

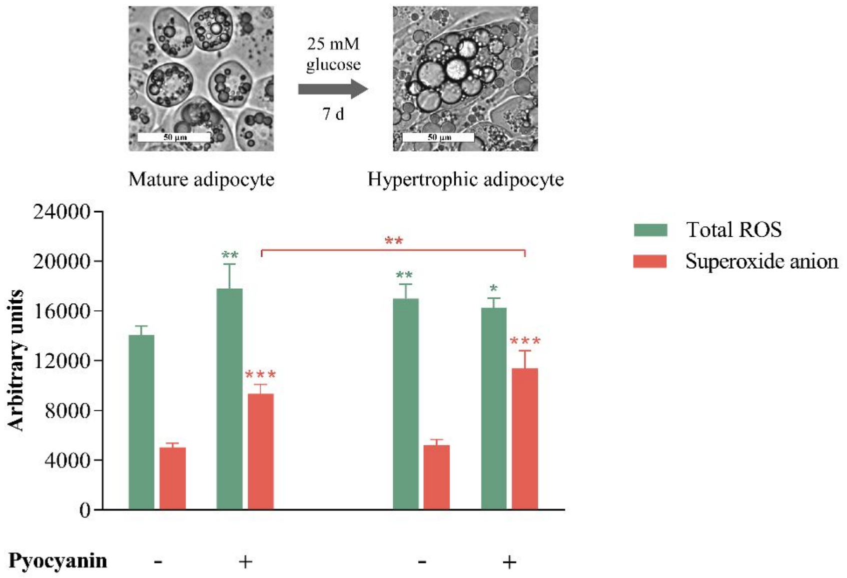

Table S1. Linear regression data, LOD and LOQ of the standard compounds monitored by DAD.

Compound	RT (min)	λ (nm)	LOD (µM)	LOQ (µM)	Linear regression equation	Correlation coefficient (r^2)
Verbascoside	33.9	340	0.15625	0.3125	y = 6.726x – 2.086	1
Isoverbascoside	36.1	340	0.15625	0.625	y = 4.305x – 3.073	0.9993
Hydroxytyrosol	16.0	280	0.625	1.5625	y = 1.607x + 0.386	0.9999
Ferulic acid	32.8	340	0.15625	0.625	y = 12.298x + 1.450	0.9999
Caffeic acid	24.3	340	0.3125	0.625	y = 6.689 – 0.282	0.9998
Homoprotocatechuic acid	16.5	280	0.625	1.5625	y = 1.596x – 0.517	0.9999
Luteolin-7-diglucuronide	26.2	340	0.15625	0.625	y = 9.388x – 6.690	0.9999

[1] RT: retention time; λ: wavelength; LOD: limit of detection; and LOQ; limit of quantification.

Figure S2. Representative chromatograms of the intracellular metabolites detected by HPLC-DAD-ESI-IT-MS in hypertrophic adipocytes after incubation with: (A) verbascoside; (B) isoverbascoside; (C) hydroxytyrosol; (D) caffeic acid; (E) ferulic acid; (F) homoprotocatechuic acid; (G) luteolin-7-diglucuronide; and (H) no compound as cellular control condition. The black lines correspond to UV chromatograms at 280 nm (C, F), 340 nm (A, B, D, E, G), or both (H). Peak numbers are those included in **Supplementary Tables S2-S8**. The colored lines show the extracted ion chromatograms at the indicated *m/z* value. The left *y*-axis indicates MS intensity, whereas the right *y*-axis specifies the UV absorption registered by DAD.

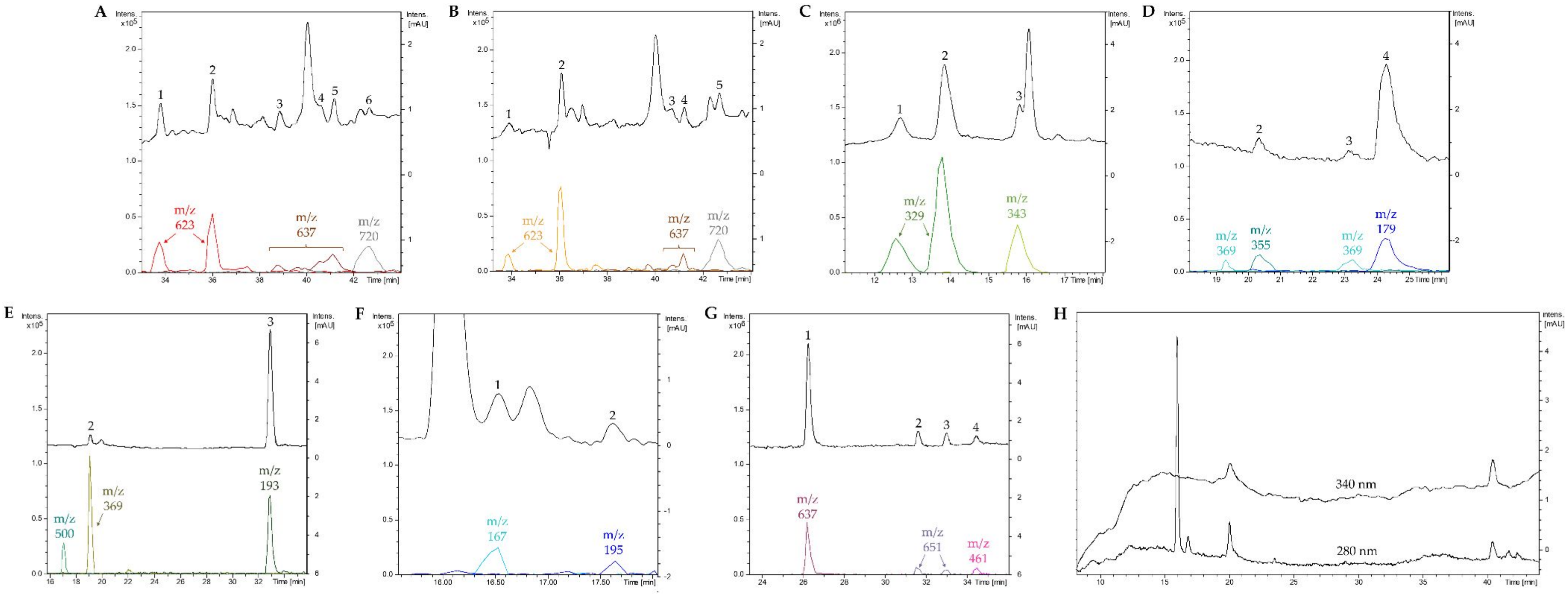

Table S2. Quantification of intracellular metabolites characterized by HPLC-DAD-ESI-IT-MS in negative mode after incubation with verbascoside in hypertrophic 3T3-L1 adipocytes. Quantification values are expressed as mean ± standard deviation (in ng of metabolite per µg of protein) (see **Supplementary Table S1** and **Figure 3**).

Peak	RT (min)	$[M-H]^-$ (*m/z*)	MS/MS (*m/z*)	Proposed metabolite	Reference	Intracellular concentration (ng/µg protein)			
						0 h	3 h	12 h	24 h
1	33.9	623	461, 315	Verbascoside	[23]	ND	0.765 ± 0.027	0.184 ± 0.009	0.182 ± 0.009
2	36.1	623	461, 315	Isoverbascoside	[23]	ND	0.580 ± 0.035	0.593 ± 0.034	0.850 ± 0.026
3	38.8	637	461, 193	Methylated verbascoside	[23]	ND	0.326 ± 0.006	0.118 ± 0.006	0.089 ± 0.004
4	40.6	637	461, 193	Methylated isoverbascoside 1	[23]	ND	ND	ND	< LOQ
5	41.2	637	461, 193	Methylated isoverbascoside 2	[23]	ND	0.342 ± 0.011	0.113 ± 0.003	0.079 ± 0.003
6	42.7	720	677, 637	Methylated isoverbascoside diacetate	-	ND	ND	0.547 ± 0.038	0.721 ± 0.058

[1] RT: retention time; ND: non detected; and LOQ: limit of quantification.

Figure S3. MS spectra of intracellular metabolites described in **Supplementary Table S2**. Each spectrum number is coincident with the peak number of the mentioned table.

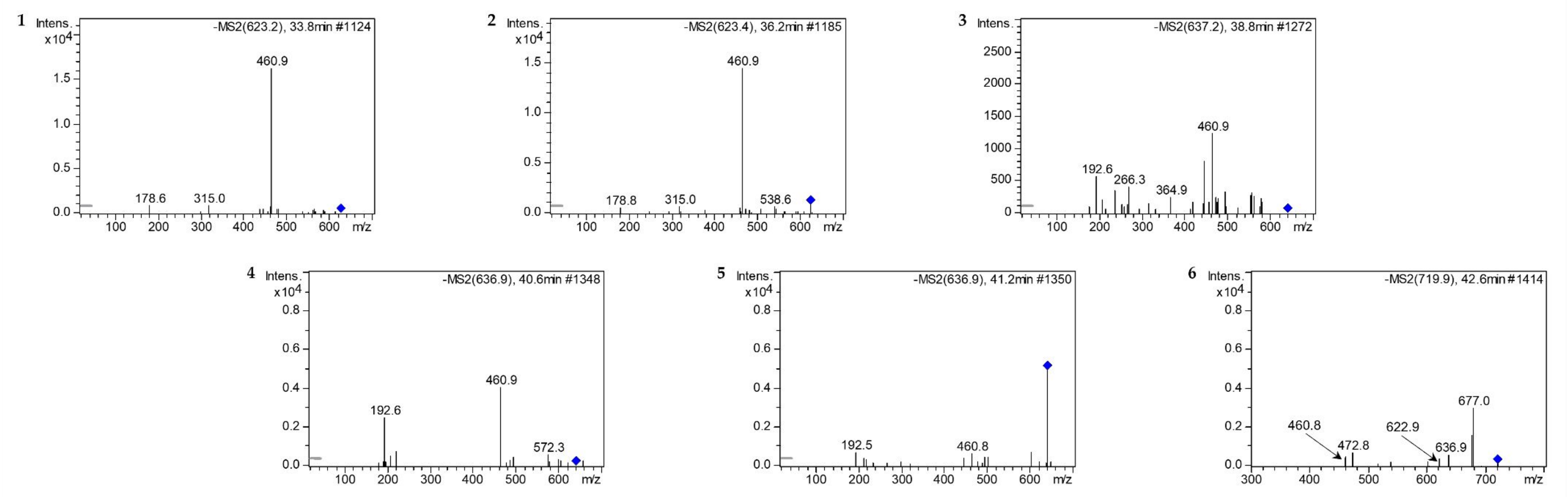

Table S3. Quantification of intracellular metabolites characterized by HPLC-DAD-ESI-IT-MS in negative mode after incubation with isoverbascoside in hypertrophic 3T3-L1 adipocytes. Quantification values are expressed as mean ± standard deviation (in ng of metabolite per µg of protein) (see **Supplementary Table S1** and **Figure 4**).

Peak	RT (min)	$[M-H]^-$ (*m/z*)	MS/MS (*m/z*)	Proposed metabolite	Reference	Intracellular concentration (ng/µg protein)			
						0 h	3 h	12 h	24 h
1	33.9	623	461, 315	Verbascoside	[23]	ND	< LOD	< LOD	< LOD
2	36.1	623	461, 315	Isoverbascoside	[23]	ND	1.541 ± 0.021	1.437 ± 0.025	1.171 ± 0.076
3	40.6	637	461, 193	Methylated isoverbascoside 1	[23]	ND	< LOQ	< LOQ	< LOQ
4	41.2	637	461, 193	Methylated isoverbascoside 2	[23]	ND	0.900 ± 0.054	0.733 ± 0.061	0.250 ± 0.015
5	42.7	720	677, 637	Methylated isoverbascoside diacetate	-	ND	ND	0.652 ± 0.062	1.127 ± 0.075

[1] RT: retention time; ND: non detected; LOD: limit of detection; and LOQ: limit of quantification.

Figure S4. MS spectra of intracellular metabolites described in **Supplementary Table S3**. Each spectrum number is coincident with the peak number of the mentioned table.

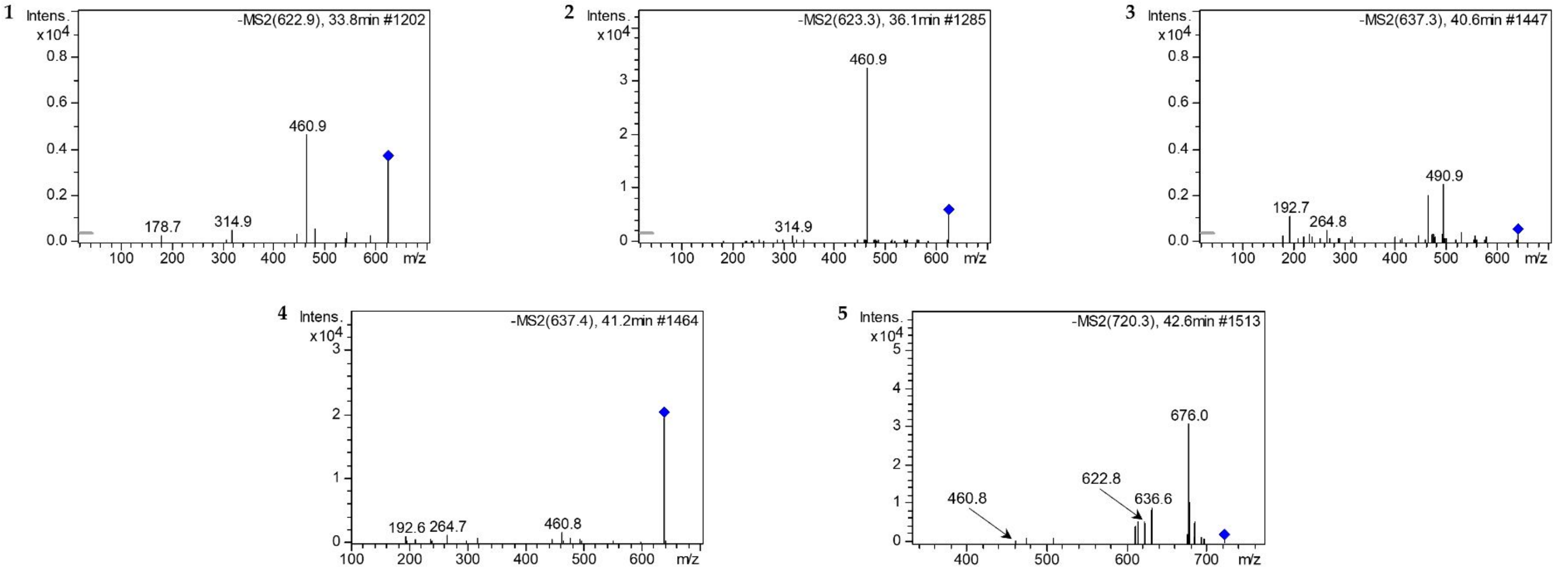

Table S4. Quantification of intracellular metabolites characterized by HPLC-DAD-ESI-IT-MS in negative mode after incubation with hydroxytyrosol in hypertrophic 3T3-L1 adipocytes. Quantification values are expressed as mean ± standard deviation (in ng of metabolite per µg of protein) (see **Supplementary Table S1** and **Figure 5**).

Peak	RT (min)	$[M-H]^-$ (*m/z*)	MS/MS (*m/z*)	Proposed metabolite	Reference	Intracellular concentration (ng/µg protein)			
						0 h	3 h	12 h	24 h
1	12.8	329	153, 123	Hydroxytyrosol glucuronide 1	[29]	ND	0.742 ± 0. 026	0.491 ± 0.044	0.222 ± 0.022
2	13.9	329	153, 123	Hydroxytyrosol glucuronide 2	[29]	ND	1.967 ± 0.029	1.024 ± 0.027	0.283 ± 0.037
3	15.8	343	167, 153	Homovanillyl alcohol glucuronide	[29]	ND	1.264 ± 0.069	1.804 ± 0.022	2.754 ± 0.040

[1] RT: retention time; and ND: non detected.

Figure S5. MS spectra of intracellular metabolites described in **Supplementary Table S4**. Each spectrum number is coincident with the peak number of the mentioned table.

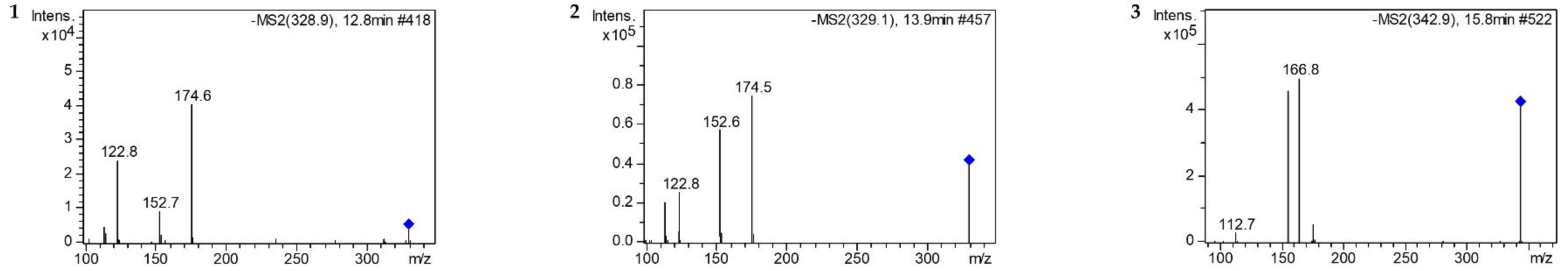

Table S5. Quantification of intracellular metabolites characterized by HPLC-DAD-ESI-IT-MS in negative mode after incubation with caffeic acid in hypertrophic 3T3-L1 adipocytes. Quantification values are expressed as mean ± standard deviation (in ng of metabolite per µg of protein) (see **Supplementary Table S1** and **Figure 6**).

Peak	RT (min)	$[M-H]^-$ (*m/z*)	MS/MS (*m/z*)	Proposed metabolite	Reference	Intracellular concentration (ng/µg protein)			
						0 h	3 h	12 h	24 h
1	19.3	369	193, 175	Ferulic acid glucuronide	[39]	ND	ND	ND	< LOD
2	20.3	355	179, 135	Caffeic acid glucuronide	[39]	ND	< LOQ	< LOQ	< LOQ
3	23.3	369	193, 175	Isoferulic acid glucuronide	[39]	ND	ND	< LOD	< LOQ
4	24.3	179	161, 135	Caffeic acid	[39]	ND	0.313 ± 0.006	0.175 ± 0.007	0.213 ± 0.015

[1] RT: retention time; ND: non detected; LOD: limit of detection; and LOQ: limit of quantification.

Figure S6. MS spectra of intracellular metabolites described in **Supplementary Table S5**. Each spectrum number is coincident with the peak number of the mentioned table.

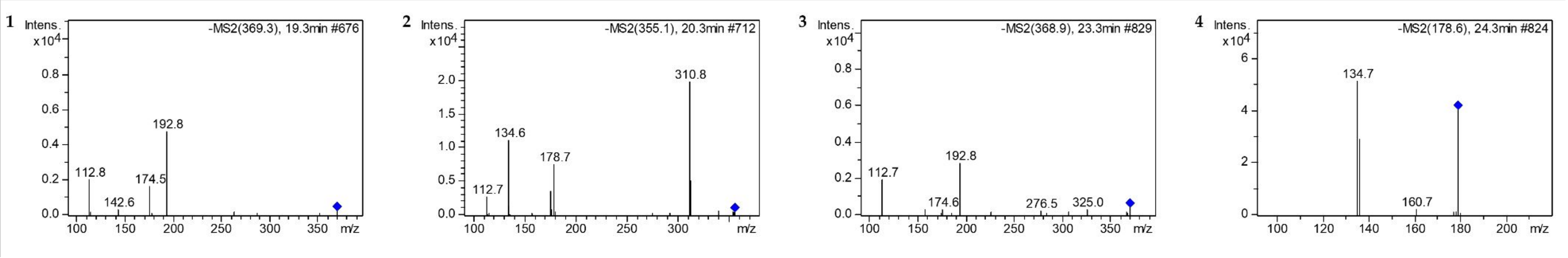

Table S6. Quantification of intracellular metabolites characterized by HPLC-DAD-ESI-IT-MS in negative mode after incubation with ferulic acid in hypertrophic 3T3-L1 adipocytes. Quantification values are expressed as mean ± standard deviation (in ng of metabolite per µg of protein) (see **Supplementary Table S1** and **Figure 7**).

Peak	RT (min)	$[M-H]^-$ (*m/z*)	MS/MS (*m/z*)	Proposed metabolite	Reference	Intracellular concentration (ng/µg protein)			
						0 h	3 h	12 h	24 h
1	17.2	500	306, 179	Unknown	-	ND	ND	ND	< LOD
2	19.2	369	193, 175	Ferulic acid glucuronide	[39]	ND	< LOQ	< LOQ	< LOQ
3	32.8	193	178, 134	Ferulic acid	[39]	ND	0.570 ± 0.022	0.515 ± 0.008	0.461 ± 0.010

[1] RT: retention time; ND: non detected; LOD: limit of detection; and LOQ: limit of quantification.

Figure S7. MS spectra of intracellular metabolites described in **Supplementary Table S6**. Each spectrum number is coincident with the peak number of the mentioned table.

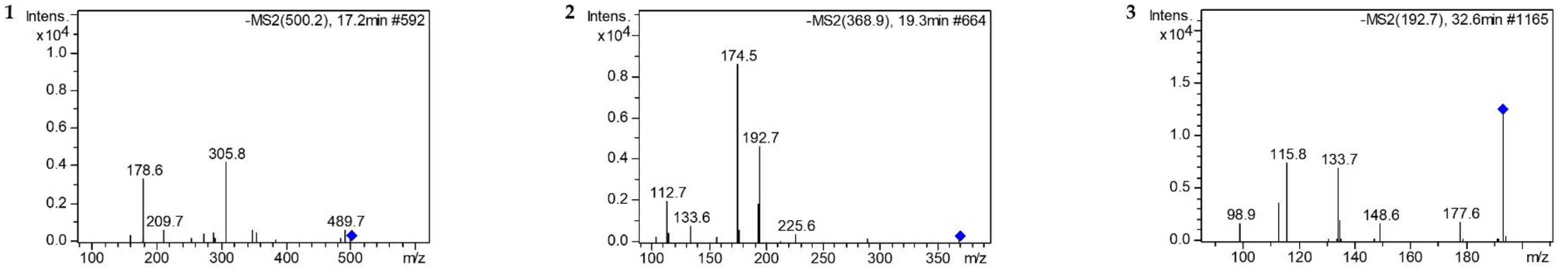

Table S7. Quantification of intracellular metabolites characterized by HPLC-DAD-ESI-IT-MS in negative mode after incubation with homoprotocatechuic acid in hypertrophic 3T3-L1 adipocytes. Quantification values are expressed as mean ± standard deviation (in ng of metabolite per µg of protein) (see **Supplementary Table S1** and **Figure 8**).

Peak	RT (min)	$[M-H]^-$ (*m/z*)	MS/MS (*m/z*)	Proposed metabolite	Reference	Intracellular concentration (ng/µg protein)			
						0 h	3 h	12 h	24 h
1	16.6	167	123, 95	Homoprotocatechuic acid	[55]	ND	0.288 ± 0.039	0.342 ± 0.015	0.187 ± 0.006
2	17.6	195	153	Homoveratric acid	[56]	ND	< LOQ	< LOQ	0.095 ± 0.005

[1] RT: retention time; ND: non detected; and LOQ: limit of quantification.

Figure S8. MS spectra of intracellular metabolites described in **Supplementary Table S7**. Each spectrum number is coincident with the peak number of the mentioned table.

1
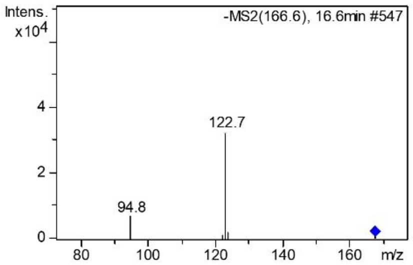

2
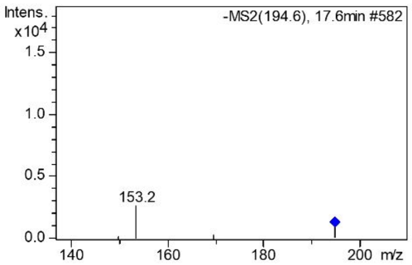

Table S8. Quantification of intracellular metabolites characterized by HPLC-DAD-ESI-IT-MS in negative mode after incubation with luteolin-7-diglucuronide in hypertrophic 3T3-L1 adipocytes. Quantification values are expressed as mean ± standard deviation (in ng of metabolite per µg of protein) (see **Supplementary Table S1** and **Figure 9**).

Peak	RT (min)	$[M\text{-}H]^-$ (*m/z*)	MS/MS (*m/z*)	Proposed metabolite	Reference	Intracellular concentration (ng/µg protein)			
						0 h	3 h	12 h	24 h
1	26.2	637	351, 285	Luteolin-7-diglucuronide	[58]	ND	1.350 ± 0.038	1.349 ± 0.058	1.359 ± 0.024
2	31.5	651	351, 299	Methylated luteolin-7-diglucuronide 1	-	ND	0.292 ± 0.004	0.273 ± 0.007	0.279 ± 0.012
3	32.9	651	351, 299	Methylated luteolin-7-diglucuronide 2	-	ND	0.283 ± 0.021	0.267 ± 0.016	0.280 ± 0.022
4	34.4	461	327, 285	Luteolin-7-glucuronide	[60]	ND	0.188 ± 0.012	0.137 ± 0.003	0.196 ± 0.012

[1] RT: retention time; and ND: non detected.

Figure S9. MS spectra of intracellular metabolites described in **Supplementary Table S8**. Each spectrum number is coincident with the peak number of the mentioned table.

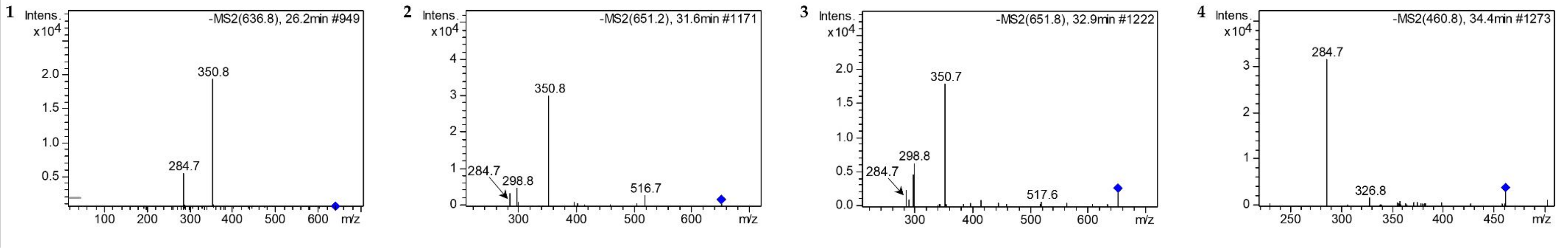

Table S9. Concentration of intracellular metabolites in the membrane fractions after incubation with verbascoside or isoverbascoside in hypertrophic 3T3-L1 adipocytes. Quantification values are expressed as a percentage with respect to the total intracellular concentration.

Incubated compound	Intracellular metabolite	Concentration in membrane (%)		
		3 h	12 h	24 h
Verbascoside	Verbascoside	27	ND	ND
	Isoverbascoside	41	38	34
	Methylated verbascoside	32	ND	ND
	Methylated isoverbascoside 2	29	ND	ND
	Methylated isoverbascoside diacetate	ND	49	48
Isoverbascoside	Isoverbascoside	40	40	36
	Methylated isoverbascoside 2	38	41	ND
	Methylated isoverbascoside diacetate	ND	47	47

[1] ND: non detected.

Table S10. Pearson correlation coefficients between the ROS levels exhibited in hypertrophic 3T3-L1 adipocytes after incubation with the seven lemon verbena compounds and the concentration of their intracellular metabolites over time. Statistical significance was determined at $p < 0.05$, with * and ** denoting $p < 0.05$ and $p < 0.01$, respectively.

Incubated compound	Intracellular metabolite	r	p
Verbascoside	Verbascoside	-0.612	0.194
	Isoverbascoside	-0.825	0.087
	Methylated verbascoside	-0.702	0.149
	Methylated isoverbascoside 2	-0.668	0.166
	Methylated isoverbascoside diacetate	-0,444	0.278
Isoverbascoside	Isoverbascoside	-0.991**	0.005
	Methylated isoverbascoside 2	-0.927*	0.037
	Methylated isoverbascoside diacetate	-0.264	0.368
Hydroxytyrosol	Hydroxytyrosol glucuronide 1	-0.639	0.181
	Hydroxytyrosol glucuronide 2	-0.490	0.255
	Homovanillyl alcohol glucuronide 1	-0.918*	0.041
Caffeic acid	Caffeic acid	-0.853	0.074
Ferulic acid	Ferulic acid	-0.937*	0.032
Homoprotocatechuic acid	Homoprotocatechuic acid	-0.944*	0.028
	Homoveratric acid	0.192	0.404
Luteolin-7-diglucuronide	Luteolin-7-diglucuronide	-0.916*	0.042
	Methylated luteolin-7-diglucuronide 1	-0.926*	0.037
	Methylated luteolin-7-diglucuronide 2	-0.915*	0.043
	Luteolin-7-glucuronide	-0.836	0.082

[1] r: Pearson correlation coefficient.
